# Transcriptomic and Metabolomics Analysis of Different Endosperm Region under Nitrogen Treatments

**DOI:** 10.3390/ijms20174212

**Published:** 2019-08-28

**Authors:** Dongyun Ma, Honghuan Gao, Chenyang Du, Lingli Li, Wan Sun, Sujun Liu, Chenyang Wang, Yingxin Xie, Guozhang Kang

**Affiliations:** 1 Agronomy College/National Engineering Research Center for Wheat, Henan Agricultural University, Zhengzhou 450046, China; 2 The National Key Laboratory of Wheat and Maize Crop Science, Henan Agricultural University, Zhengzhou 450046, China

**Keywords:** storage proteins, innermost endosperm region, remaining endosperm region, metabolome, transcriptome, wheat

## Abstract

Storage protein distribution in wheat-grain endosperm is heterogeneous, but the underlying molecular mechanism remains unclear. Two parts of the endosperm region, the innermost endosperm (IE) region and the remaining endosperm (RE) region, grown under low nitrogen (LN) and high nitrogen (HN) treatments were used to perform metabolomic and transcriptomic analysis. We identified 533 and 503 differentially expressed genes (DEGs) with at least a two-fold expression change (*p* < 0.05) between IE and RE, among which 81 and 78 transcripts under LN and HN, respectively, related to carbon and nitrogen metabolism, and encoded transcription factors or proteins involved in post-translational modification (PTM). The significantly differentially abundant metabolites between IE and RE were mainly amino acids, N-compounds, carbohydrates, and nucleic acids. More upregulated transcripts and metabolites were identified in RE than IE under HN conditions, indicating that HN activates metabolism in the endosperm periphery. In addition to carbon and nitrogen metabolism, transcription factors and protein PTMs, such as phosphorylation and acetylation, might determine the protein heterogeneous distribution between IE and RE and its response to nitrogen fertilizer supply.

## 1. Introduction

Proteins are one of the main components of wheat grain starchy endosperm and have been used as an index to predict and evaluate flour quality for end products [1]. The qualitative and quantitative distribution of grain storage protein (GSP) in the endosperm occurs as a gradient and is heterogeneous [2,3]. It is generally believed that the protein content is lower in the inner parts of the endosperm and increases in an outwards radial direction [4]; whereas protein quality increases form the outer to the inner endosperm [2]. Savill et al. [5] found that protein is concentrated in the endosperm nearest to the aleurone layer and decreases in an inwards direction to the center of the two lobes. According to Chen et al. [6], dorsal endosperm tissues had higher protein content in comparison with abdominal endosperm tissues. Heterogeneous distribution in wheat endosperm exists in two components of glutenins; high-molecular-weight glutenin subunits (HMW-GS) and low-molecular-weight glutenin subunits (LMW-GS). HMW-GS are expressed more highly in the inner endosperm and LMW-GS are more abundant in the subaleurone layer [5]. Zhou et al. [7] produced nine tissue layers by the pearling milling method and found that the contents of HMW-GS and glutenin macropolymers got the highest value at the second or the third layer. The inhomogeneity in protein quantity and quality in wheat endosperm produce flour with different protein contents and compositions, which influence food quality [7,8].

Protein distribution heterogeneity in the concentration and quality within the wheat endosperm are generally accepted to exist [2,5], although the underlying mechanism for its generation remains to be clearly described. Ugalde and Jenner [9] reported that the amino acid substrate supply and transport pattern across the endosperm does not limit the protein deposition. Protein distribution heterogeneity might be attributable to the strong sink activity of the subaleurone cells, which drive amino acid transport from the endosperm cavity cells to the subaleurone layer [10] or to the size of protein bodies, which decreases from the outer to the inner endosperm layers [5]. Previous studies also showed that nitrogen uptake by plants at different developmental stages also affected protein components in different layers of grain endosperm [11]. Tosi et al. [2] speculated that the protein inhomogeneity distribution might be due to the different transcription rates of gluten protein genes that are potentially regulated by differentially expressed specific transcription factors or regulatory signals.

Nitrogen fertilization is one of the important agronomic measures for wheat production and an appropriate fertilization rate can increase wheat grain yield and improve grain quality [12,13,14]. Recent results have shown that the level of nitrogen fertilizer application also determines the steepness of the heterogeneity in protein content and quality in the endosperm [3,5]. Increasing the nitrogen supply increased the HMW-GS content, but this increase was greater in the outer endosperm than near the central endosperm, whereas the sulfur-rich prolamins showed an opposite change in response to nitrogen fertilizer [3]. The nitrogen fertilizer application also affected the synthesis and accumulation of ω-gliadin within the endosperm; in response to increased nitrogen fertilizer application, more ω-gliadin concentrated in the outer endosperm layers [15]. Furthermore, modern commercial wheat flour mills produce white flour via a milling and sieving process and a proportion of endosperm protein that adheres to the aleurone and bran layer is lost with bran removal. Thus, the increased steepness of the protein heterogeneity in the endosperm following nitrogen application results in more protein being lost during the production of white flour [5]. The underlying mechanism of the effect of nitrogen fertilization on grain protein content has been investigated. The increased concentration of ω-gliadins in the subaleurone layer induced by the increasing nitrogen supply might result from the high expression of genes upregulated by nitrogen application [15]. Zhang et al. [14] suggested that high nitrogen (HN) treatment could increase grain yield and protein content by improving the expression levels of genes related to nitrogen metabolism. Yu et al. [16] reported that high HN treatment increases protein aggregation by improving—peptidyl-prolyl cis–trans isomerase (PPIase) SUMOylation with the assistance of small ubiquitin-related modifier1 (SUMO1). Previous studies also showed that grain protein synthesis has been regulated by transcription factors [17,18]. Due to the pressure of the future population explosion and environmental pollution caused by increasing excessive nitrogen fertilizer application, limiting the application of nitrogen fertilizer to increase yield and improve grain quality is particularly important. To elucidate the underlying molecular mechanism of the heterogeneity of protein content between different parts of endosperm and its response to nitrogen treatment, we divided grain endosperm into two parts, the innermost endosperm region and the remaining region, and characterized the transcriptional and metabolic differences between these two parts in plants grown in low or high nitrogen conditions. This study provides insights into the heterogeneity of the inner and outer endosperm proteins and their response to the application of nitrogen fertilizer.

## 2. Results

### 2.1. Grain Characteristics 

Wheat plants grown under HN conditions had a higher grain yield than grains with the low nitrogen (LN) treatment (Table 1). The remaining endosperm (RE) region possessed a higher protein and component content than the innermost endosperm (IE) region in both HN and LN treatments. The protein content of RE and IE was higher in the HN treatment than in the LN treatment, but the protein content in RE increased to a greater extent than that in the IE (21 mg g^−1^ vs. 17 mg g^−1^). By contrast, the content of gluten (gliadin and glutenin) was higher in the IE region than in RE (6.3 mg g^−1^ vs. 3.3 mg g^−1^). The data confirm that RE regions had a higher protein content than IE. Increasing the nitrogen fertilizer supply can therefore enhance grain protein content, especially in the outer endosperm.

### 2.2. Transcriptome Analysis of the Innermost Endosperm Region and the Remianing Endosperm Region from Plants Grown at Two Nitrogen Fertilizer Levels

The IE and RE regions in the developing wheat grain under the HN and LN fertilizer treatments were separated, and 12 complementary DNA (cDNA) libraries were constructed. After sequencing, the uniquely mapped reads ranged from 37,768,733 to 41,460,651, representing 67.4–70.0% of the total reads (Table S1). The number of non-spliced reads ranged from 30,794,313 to 32,898,691, representing 53.4–55.6% of the total reads. The number of uniquely mapped reads and non-spliced reads in the IE region (LN-IE and HN-IE) was lower than in the RE region (LN-RE and HN-RE).

**Figure 1:**
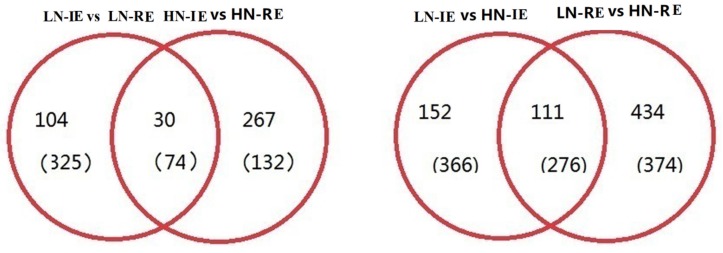
Ven diagram of differentially expressed genes in different parts of wheat grain endosperm under high and low nitrogen treatments. HN-IE and HN-RE are samples from innermost endosperm region and the remaining endosperm region under high nitrogen treatment, respectively. LN-IE and LN-RE are samples from innermost endosperm region and the remaining endosperm region under low nitrogen treatment, respectively. The number outside (inside) brackets stand for up-regulation (down-regulation) differentially expressed genes.

In total, 533 and 503 genes were significantly differentially expressed between LN-IE and LN-RE and between HN-IE and HN-RE, respectively (Figure 1). An additional 905 genes were significantly differentially expressed in IE between HN and LN treatments, whereas 1195 genes were detected in RE, which indicates that increasing the nitrogen fertilizer application rate more specifically affected gene expression in RE than in the IE. The enriched Gene Ontology (GO) terms showed that the differentially expressed genes (DEGs) identified in this study involved in biological process were categorized into 14 groups, including hydrolase activity, a oxidation-reduction process, a catabolic process, biological regulation, and a response to chemicals (Supplementary Figure S1). The molecular functions mainly involved oxido-reductase activity (12.11–17.39%), hydrolase activity (18.95–25.30%), and transferase activity (16.60–18.84%). The cell component mainly included a cell part (29.38–32.17%), an integral component of membrane (17.66–25.40%), and intracellular (22.75–27.06%) categories.

A carbon/nitrogen metabolism is crucially important for wheat-grain protein synthesis. To investigate the role of carbon/nitrogen metabolism in the difference in the storage protein content between IE and RE, we identified the DEGs associated with carbon/nitrogen metabolism in Supplementary Table S2. In total, 32 DEGs involved in nitrogen metabolism were detected between LN-IE and LN-RE (Supplementary Table S2-I). Compared with the remaining endosperm part under LN treatment (LN-RE), 15 genes were upregulated in the LN-IE part, including those encoding two nitrate reductases (TraesCS6A01G017500 and TraesCS6D01G020700), two glutathione transferases (TraesCS1A01G153100 and TraesCS3A01G302100), and five serine-type endospeptidases (TraesCS4B01G077600, TraesCS4D01G076000, TraesCS5A01G526000, TraesCS1D01G395200, and TraesCS3B01G515100). The expression levels of the nitrate reductase-encoding genes in LN-IE were 2.14- and 3.61-fold greater than that in the LN-RE region. Additionally, the upregulated genes in the LN-RE region encoded four carboxypeptidases (TraesCS1A01G086100, TraesCS1D01G087600, TraesCS1B01G104500, and TraesCS5D01G196500) and five amino acid transmembrane transporters (TraesCS2B01G551300, TraesCS2A01G348600, TraesCS7A01G194500, TraesCS7B01G100100, and TraesCS7D01G196100). The expression levels of these genes in the LN-RE region were approximately 1.78–2.92-fold greater than that in the LN-IE regions, indicating that amino acid production and transport are more active in the peripheral endosperm region. Correspondingly, 29 DEGs involved in nitrogen metabolism were detected between HN-IE and HN-RE (Supplementary Table S2-II), including 18 upregulated transcripts in the innermost endosperm part in comparison with the endosperm peripheral region. The upregulated genes in the HN-IE sample encoded two serine-type endospeptidases (TraesCS1A01G188900 and TraesCS4D01G076000), one gliadin (TraesCSU01G153800), and three serine protease inhibitors (TraesCS5D01G425800, TraesCS5B01G478300, and TraesCS5A01G359700). However, the upregulated genes in the HN-RE region contained genes encoding two aspartic-type and one cysteine-type peptidase (TraesCS2D01G109600, TraesCS3D01G467300, and TraesCS7D01G060100), two globulins (TraesCS7D01G351300 and TraesCS4D01G171800) and one ammonium transmembrane transporter (TraesCS1D01G296600), which was expressed 4.52-fold higher than that in HN-IE. Considering DEGs involved in carbon metabolism, the LN-IE vs. LN-RE comparison revealed 18 DEGs, including 10 that were upregulated in LN-IE and in HN-IE vs. HN-RE and 28 DEGs, included 20 that were upregulated in HN-IE. Among the upregulated transcripts, those in the RE region were involved in glycoside hydrolysis, and those in the IE region were glycoside transferases. A comparison between the genes expressed in the HN and LN treatments revealed that 17 and 51 DEGs involved in nitrogen metabolism were detected in IE and RE, respectively (Supplementary Table S2-III, S2-IV). These results suggest that increasing the nitrogen fertilizer supplying level might activate nitrogen metabolism, especially in the outer endosperm. The HN treatment induced 13 genes in IE, including six endopeptidase inhibitors, two protease inhibitors, and two protein transporters. In the RE region, 39 genes were upregulated by HN treatment, including three transmembrane transporters. Furthermore, most of the upregulated genes induced by HN were involved in promoting (including 10 encoding proteins with peptidase activity) or inhibiting (including 13 encoding peptidase inhibitors) protein degradation. These results indicate that HN treatment induces complex and active regulatory nitrogen metabolism processes.

**Figure 2:**
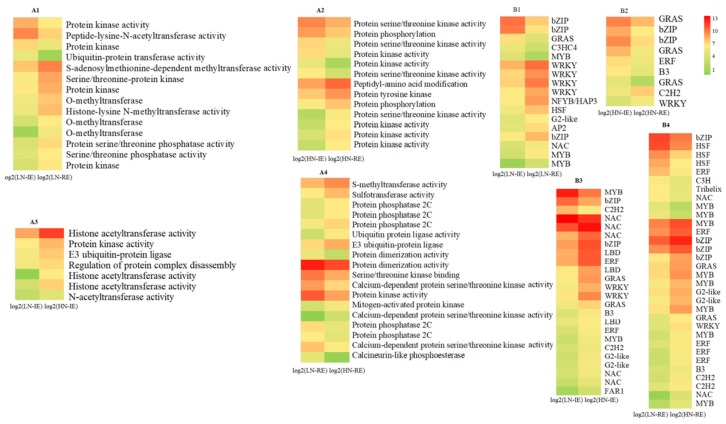
Heat map of differentially expressed transcription factor genes, and transcripts involved.in protein phosphorylation, methylation and ubiquitination. HN-IE and HN-RE are samples from innermost endosperm region and the remaining endosperm region under high nitrogen treatment, respectively. LN-IE and LN-RE are samples from innermost endosperm region and the remaining endosperm region under low nitrogen treatment, respectively. (**A1**–**A4**) indicate the genes involved in protein post-translation modification. (**B1**–**B4**) indicate the transcription factor genes. Red color represent high expression level and green represent low expression level.

Post-translational modifications play a key role in functional proteins and are usually used to regulate cellular activity. Here, 14 DEGs associated with protein phosphorylation, methylation, and ubiquitination were detected in the LN-IE vs. LN-RE comparison, including 10 upregulated in RE (Figure 2A1). Correspondingly, six upregulated genes involved in post-translation modification were detected in the HN-RE sample (Figure 2A2). Correspondingly, 7 and 18 genes were annotated in LN-IE vs. HN-IE (Figure 2A3) and in LN-RE vs. HN-RE (Figure 2A4), respectively. Most of the DEGs in the IE region were involved in histone acetylation and those in the RE region were related to protein phosphorylation, suggesting that different regulatory mechanisms in the inner and outer endosperm regions might exist in response to increasing nitrogen supply.

In the LN-IE vs. LN-RE comparison, 17 DEGs mapped to the transcription factor category (Figure 2B1). Five were upregulated in LN-IE, including two that encoded basic leucine zipper (bZIP) transcription factors, whereas 12 DEGs were upregulated in LN-RE, including genes encoding four WRKY- and two MYB-type transcription factors. However, nine DEGs were identified in the HN-IE vs. HN-RE comparison, seven of which were upregulated in HN-IE (Figure 2B2). Genes encoding two transcription factors (TraesCS2B01G217500, GRAS; TraesCS7B01G391800, bZIP) were significantly upregulated in the IE region in both HN and LN treatments. Among the 24 differentially expressed genes that encoded transcription factors in LN-IE vs. HN-IE, 20 were upregulated by HN treatment (Figure 2B3). Correspondingly, 32 DEGs encoding transcription factors were detected in the LN-RE vs. HN-RE comparison, among which 23 genes were upregulated by HN treatment (Figure 2B4), and most of these upregulated genes encoded transcription factors from the bZIP, ethylene-responsive factor (ERF), and MYB families. These results suggest that high nitrogen availability activates transcription. Furthermore, among the differentially expressed transcription factors induced by increased nitrogen supply, 11 were expressed in both the IE and RE regions.

### 2.3. qRT-PCR and BSMV-VIGS Validation of Identified Genes

**Figure 3:**
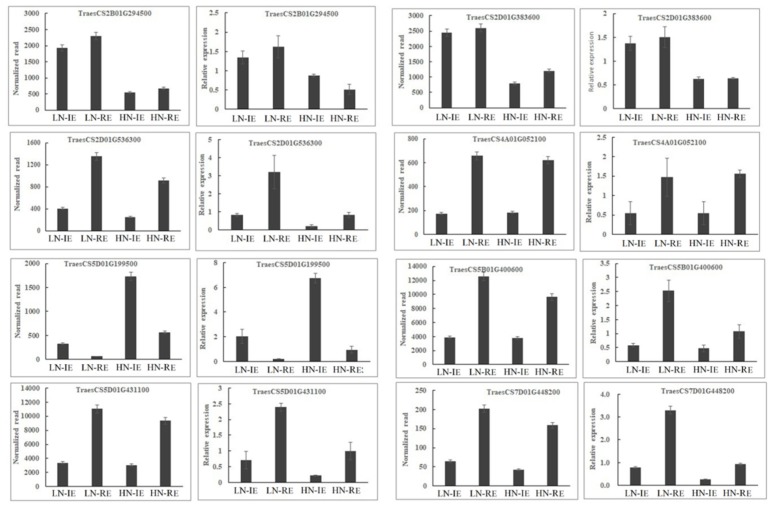
Verification of expression levels for eight differentially genes in the inner and outer endosperm grown under high or low nitrogen conditions. HN-IE and HN-RE are samples from innermost endosperm region and the remaining endosperm region under high nitrogen treatment, respectively. LN-IE and LN-RE are samples from innermost endosperm region and the remaining endosperm region under low nitrogen treatment, respectively.

**Figure 4:**
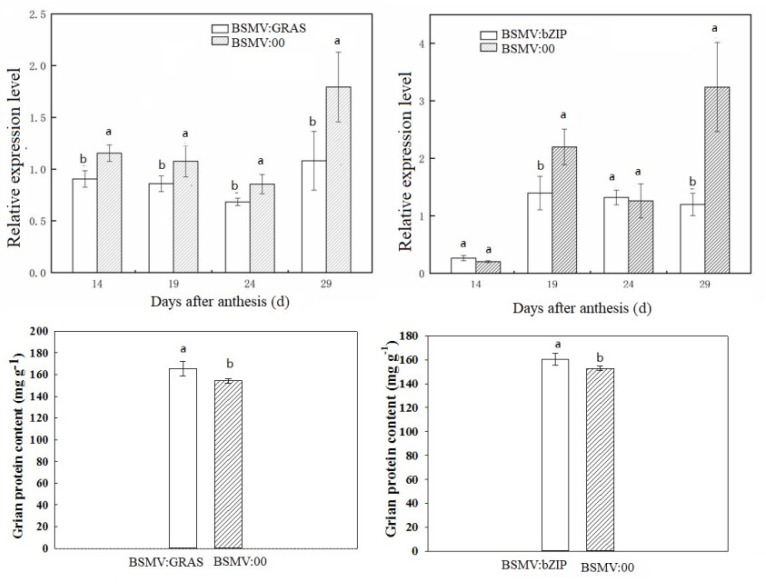
Gene functional analysis by BSMV-VIGS. BSMV-VIGS, barley stripe mosaic virus-based virus-induced gene silencing system; BSMV:00 indicate empty vector; BSMV:bZIP and BSMV:GRAS indicate that silencing bZIP and GRAS gene, respectively; Different lowercase letters above the column on the same day indicate a significant difference (*p* < 0.05).

Transcript expression levels were confirmed by qRT-PCR. The expression patterns of eight randomly selected genes were similar to those obtained from deep-sequencing (Figure 3), confirming the reliability of the observed transcript levels. The barley stripe mosaic virus (BSMV)-based virus-induced gene silencing (VIGS) has been used as an effective method for evaluating gene functions in wheat. Genes encoding two transcription factors (TraesCS7B01G391800, *TabZIP1;* TraesCS2B01G217500, *TaGRAS*) were used to validate its function by the BSMV-VIGS method. Here, the BSMV:bZIP and BSMV:GRAS vectors were constructed and used to inoculate wheat spikes at the booting stage, with BSMV:00 as the empty vector control. The expression levels of *TabZIP1* and *TaGRAS* in induced grain were significantly lower than in the control spike, which indicates that their expression was successfully suppressed (Figure 4). Furthermore, the storage protein content in grain from the induced spike was higher than that in the control grains, suggesting that *TabZIP1* and *TaGRAS* might negatively regulate storage protein deposition.

### 2.4. Metabolic Profiling of the Innermost Endosperm Region and the Remaining Endosperm Region under LN and HN Conditions

**Figure 5:**
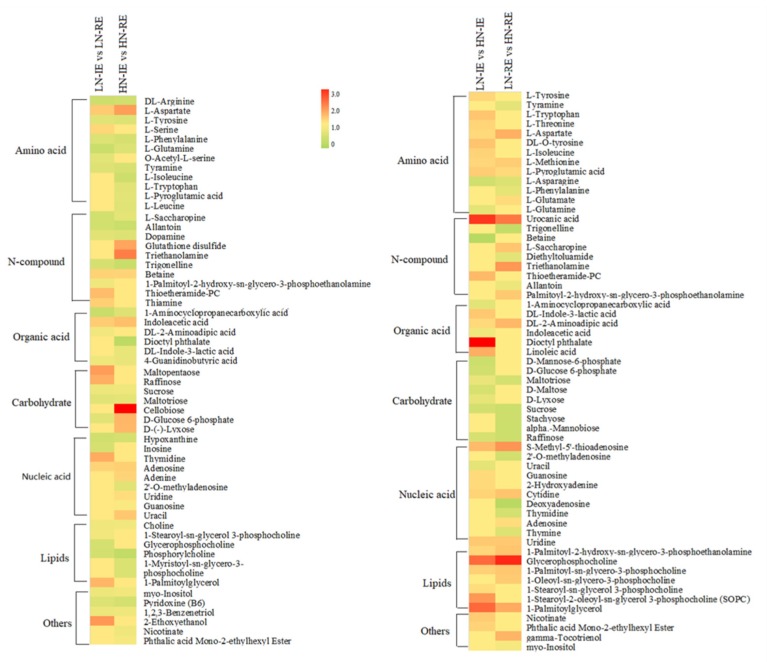
Heat map of differentially expressed metabolites in two parts of endosperm region under high and low nitrogen treatment. HN-IE and HN-RE are samples from innermost endosperm region and the remaining endosperm region under high nitrogen treatment, respectively. LN-IE and LN-RE are samples from innermost endosperm region and the remaining endosperm region under low nitrogen treatment, respectively. Heat map represent the ration of relative abundance of LN-IE vs LN-RE, HN-IE vs HN-RE, LN-IE vs HN-IE, and LN-RE vs HN-RE.

Metabolic profiling of the IE and RE regions from LN and HN treatments was conducted using gas chromatography-mass spectrometer (GC-MS). Metabolites that showed significant differences in abundance between these two parts of endosperm are showed in Figure 5. The major metabolites were amino acids, N-compounds, organic acids, carbohydrates, and lipids. Most metabolites were present at higher concentrations in the RE region, indicating a more active metabolism in the peripheral endosperm region. The level of most of the differentially expressed metabolites involved in amino acid and N-compounds increased by the LN treatment, whereas carbohydrate metabolites showed a higher level in the HN treatment. 

The distribution of all significantly differentially expressed metabolites was analyzed by principal component analysis (PCA) (Figure S2). The metabolites identified in the same treatment clustered together. The difference in metabolite levels between the IE and RE regions was clearly distinguished by the first principal component (PC1), and the second principal component (PC2) clearly separated HN and LN samples. We further analyzed the PCA loading scores, which showed that the most important metabolites relating to the difference between IE and RE were three amino acids, three N-compounds, one carbohydrate, and one nucleic acid (Supplementary Table S3). Additionally, the important metabolites that contributed to the difference between the HN and LN treatment were two amino acids (L-methionine, L-pyroglutamic acid), one organic acid (DL-2-aminoadipic acid), two N-compounds (thioetheramide-PC, 1-Palmitoyl-2-hydroxy-sn-glycero-3-phosphoethanolamine), two carbohydrates (raffinose, cellobiose), and one lipid (glycerophosphocholine).

**Figure 6:**
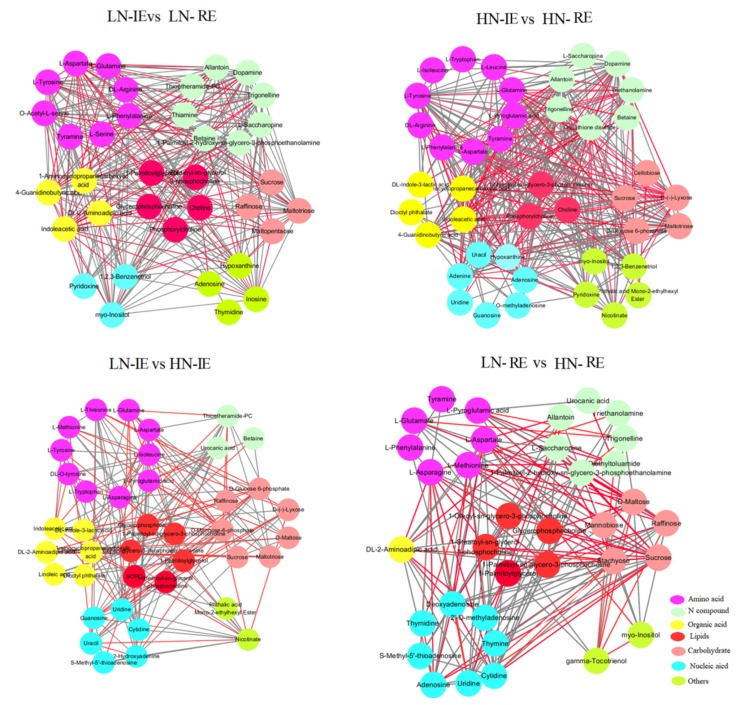
Network of metabolite-metabolite correlation based on significant correlations. Nodes stand for metabolites and edges stand for metabolite relationship. Red edges mean negative correlation, and grey edges mean positive correlation. The nodes in different color mean different metabolite type. HN-IE and HN-RE are samples from innermost endosperm region and the remaining endosperm region under high nitrogen treatment, respectively. LN-IE and LN-RE are samples from innermost endosperm region and the remaining endosperm region under low nitrogen treatment, respectively.

The relationships between these metabolites were analyzed by pairwise correlations. The network of significantly correlated (*r*^2^ ≥ 0.50) metabolites was drawn using Cytoscape v.3.6.0 (www.cytoscape.org/). In total, 36 metabolites and 263 correlations were detected in the LN-IE vs. LN-RE group and 42 metabolites and 285 correlations in the HN-IE vs. HN-RE group (Figure 6). Additionally, 19 and 24 correlations were identified among the tested amino acids in the LN and HN samples, respectively. These data demonstrate that a high nitrogen supply activates metabolism in the endosperm. Positive correlations were observed between tyramine, L-tyrosine, and phenylalanine, and most of the correlations between these amino acids and carbohydrates, N-compounds, and organic acids were also positive. However L-aspartate was highly negatively correlated with other metabolites (organic acids, carbohydrates, and lipids). A positive relationship was observed between O-acetyl-L-serine and other amino acids, N-compounds, and carbohydrates, but no direct correlation was observed with lipids under LN treatment. Among the significantly different metabolites, 138 and 139 correlations were identified in the LN-IE vs. HN-IE group and the LN-RE vs. HN-RE comparisons, respectively (Figure 6).

### 2.5. Combined Transcriptome and Metabonomics Analysis

A comparative analysis of kyoto encyclopedia of genes and genomes (KEGG) pathway enrichment was performed between the transcriptome and metabolome. Three pathways were enriched between LN-IE and LN-RE, including starch and sucrose metabolism, nitrogen metabolism, and glyoxylate and dicarboxylate metabolism (Figure S3A). Correspondingly, two KEGG pathways were also enriched between the HN-IE and HN-RE group, including starch and sucrose metabolism and galactose metabolism (Figure S3B). These results suggest that carbon and nitrogen metabolism are important in determining a non-homogeneous protein distribution between the inner and outer endosperm region. 

**Figure 7:**
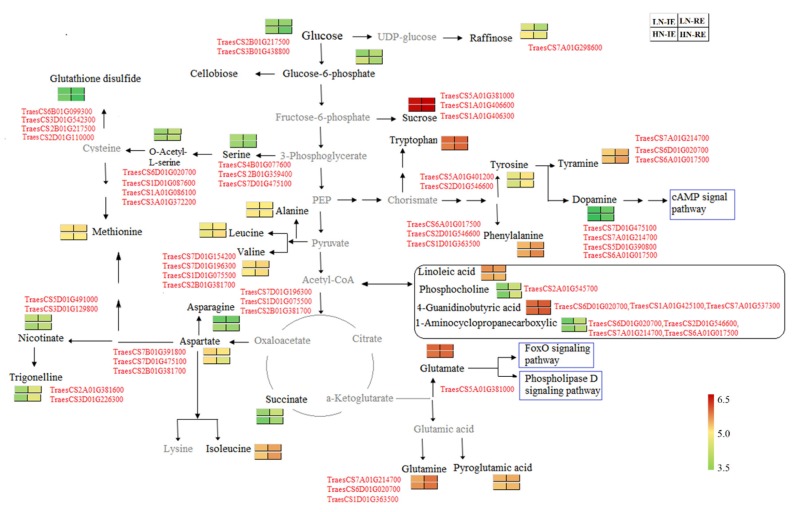
Schematic map of metabolic pathway involved in different regions of wheat grain endosperm grown under high nitrogen and low nitrogen conditions. The 2 × 2 heat map represent normalized (log10) relative metabolites abundance at different regions of endosperm under high and low nitrogen treatment with red color for a higher abundance and green color for a lower abundance. HN-IE and HN-RE are samples from innermost endosperm region and the remaining endosperm region under high nitrogen treatment, respectively. LN-IE and LN-RE are samples from innermost endosperm region and the remaining endosperm region under low nitrogen treatment, respectively. Metabolite in black color represent identified metabolite in this study.

The correlation coefficients between DEGs and significant differentially expressed metabolites were calculated by the methods of Spearman. The correlation network was plotted using a correlation coefficient |*r*| ≥ 0.5 and *p* < 0.01, using Cytoscape (Supplementary Figures S4 and S5). Thiamine, tyramine, dopamine, tyrosine, and phenylalanine were regulated (positively and negatively) by a group of similar genes. O-acetyl-L-serine and trigonelline correlated positively with five genes. A sketch map was drawn to direct the metabolic pathways involved in the difference between IE and RE (Figure 7). Genes encoding nitrate reductase (TraesCS6D01G020700 and TraesCS6A01G017500) and transcription factors (TraesCS5A01G101200, TraesCS7A01G214700, and TraesCS7D01G475100) correlated positively with tyrosine, tyramine, and phenylalanine. The significantly differentially expressed metabolites, such as serine, O-acetyl-L-serine, and glutathione disulfide, suggest that cysteine and methionine metabolism is involved in establishing the protein distribution heterogeneity in wheat-grain endosperm. The positive correlation between serine-type endopeptidase (TraesCS4B01G077600), a bZIP transcription factor (TraesCS7D01G475100) and serine, protein kinase (TraesCS3D01G542300), transcription factors (GRAS and ERF), and glutathione disulfide indicate that, in addition to nitrogen metabolism, protein post-translational modifications and transcription factors might also regulate protein deposition in wheat-grain endosperm.

## 3. Discussion

Because of the structure of the endosperm, it is not easy to completely separate the interior layer endosperm tissue from the endosperm peripheral region. Micro-dissection [9,19], microscopy [5,6], pearling milling [7], and immunofluorescence [2] have been used to study qualitative and quantitative protein gradient distributions in wheat-grain endosperm. Here, we manually divided the middle grain segment into two parts, the innermost endosperm part and the remaining endosperm part, and analyzed the difference in protein content between these endosperm parts under HN and LN treatment. The results showed that the protein distribution heterogeneity between the RE and IE regions was greater in the HN treatment than under low nitrogen availability, which is consistent with the data of Savill et al. [5]. Previous studies reported that protein quality is also distributed in non-homogeneity [20] and that flour fractions from the central endosperm generally have better dough functional properties than those from the outer endosperm [7,21]. In this study, the protein content in the RE region increased (21 mg^−1^) more than that in the innermost endosperm region (17 mg g^−1^) following the application of nitrogen fertilizer, whereas the gluten content increased more in the IE region than in the RE region, which indicates a differential response of protein quantity and quality to nitrogen fertilizer treatment in different parts of wheat grain endosperm. He et al. [3] also reported a similar disproportionate increase in protein components in different endosperm regions following an increased nitrogen supply. Li et al. [11] found that the contribution of nitrogen assimilated at different developmental stages to grain protein fractions varied among different layers of endosperm; for glutenin, the contribution of N assimilated after anthesis showed a decrease tendency from the outer layer to the inner layer. Further information concerning the quality of flour derived from different parts of wheat endosperm grown under different nitrogen fertilizer treatments is required.

It was speculated that the endosperm protein heterogeneity is attributable to specific transcription factors or other signals that regulate the transcription levels of genes related to gluten biosynthesis [6,20]. It has been suggested that translation and/or post-translational regulation regulates grain storage-protein synthesis [22,23]. Here, approximately 500 DEGs were detected between the IE and RE regions, which suggests that transcriptional regulation is important in the formation of protein heterogeneous distribution. Although endosperm protein content distribution was not attributed to the pattern of starch deposition [19], the content of starch also decreased from the inner to the outer endosperm [7]. Carbon metabolism is the most important metabolic process for plant growth, development, and yield quality. In this study, more genes involved in carbon metabolism, mainly those encoding glycoside hydrolases, were upregulated in the IE region than in the RE region, especially in high nitrogen conditions. In addition, the content of several sugars, such as mannose-6-phosphate, sucrose, and maltotriose, increased, following the high nitrogen treatment, consistent with the findings of Zhen et al. [24]. These differentially expressed metabolites might contribute to grain yield and quality, because carbohydrates not only represent a major energy store, but provide essential structural carbon skeletons [25]. Previous studies on the functional validation of key genes (*AGPase* and *HvSUT1*) involved in carbon metabolism showed that manipulating carbon metabolism also affected the synthesis of GSP [26,27].

Nitrogen metabolism is critically important for wheat GSP accumulation and it has been suggested that the activity of several enzymes, including nitrate reductase, glutamine synthetase, and glutamate synthase, is correlated with protein content [14]. We found that genes encoding two nitrate reductases were significantly more highly expressed levels in the IE region compared with the RE region. Li et al. [28] found a medium negative correlation between nitrate reductase activity (NRA) and grain protein content. Here, the opposite relationship between nitrate reductase gene expression (TraesCS6A01G017500 and TraesCS6D01G020700) and protein content between IE and RE agrees with Li et al. [28], indicating that nitrogen assimilation is important for the protein distribution difference. Protein degradation and biosynthesis occur throughout the complete plant life-cycle and play an important role in plant growth and development [29]. Protein degradation promotes the turnover and reuse of amino acids; however, some protein degradation products might also perform a signaling role [29,30,31]. Protein degradation requires the participation of a variety of proteolytic enzymes, including endopeptidases, aminopeptidases, and carboxypeptidases [32]. In this study, more transcripts encoding serine-type endopeptidases were upregulated in the IE region and the genes encoding carboxypeptidases, aspartic- or cysteine-type endopeptidases were upregulated in the RE region, indicating that different protein degradation processes might characterize the IE and RE regions. The metabolomic analysis here also demonstrated a higher content of amino acids and N-compounds in the RE region rather than the IE region in both HN and LN treatments. Furthermore, most identified DEGs relating to transmembrane amino acid transporters were upregulated in the RE region. These results suggest that increased amino acid turnover and transport might promote protein deposition in the outer endosperm. Ugalde and Jenner [9] suggested that amino acid transport across the endosperm does not limit protein deposition in the endosperm, but they also speculated that differential glutamine supply might explain the difference in protein deposition between the outer and inner endosperm. Here, the higher glutamine content in the remaining endosperm region might confirm that the amino acid supply relates to the high protein deposition in the outer endosperm region. A close relationship between amino acid transporters and GSP content has been reported in wheat and barley [23,33]. Clearly, the increased amino acid content in the innermost endosperm region might function in other regulatory mechanisms related to storage protein deposition, because it has been shown that amino acid precursors, such as O-aceylserine, regulate the GSP content [29,34].

Transcription factors regulate gene transcription. Previous results suggested that several transcription factors, such as *MYBS3* and *FUSCA3*, might be involved in regulating GSP [35,36]. Here, several transcription factors, including bZIP, MYB, WRKY, APETALA 2 (AP2), and NAM/ATAF/CUC (NAC) family members, were differentially expressed between IE and RE, with most of the bZIP and GRAS transcription factors being upregulated in the IE region, and WRKY and MYB proteins being upregulated in the RE region. In rice, *RISBZ1*/*OsbZIP58* regulated GSP synthesis [37,38], whereas Yang et al. [39] found that reducing the expression of *TaZIP60* increased the wheat yield and N-use efficiency. After sequence alignment, we found here that TraesCS6D01G312800 shared 99.5% identity with *TaZIP60,* with one amino acid difference and one absence. In this study, the HN-RE sample showed a high expression level of *TaZIP* (TraesCS6D01G312800), but had high protein content. This difference might be partially due to the different plant organs studied (root vs. grain) or due to the amino acid sequence differences. It has been found that many transcription factors belong to multigene families with diverse functions in plant growth and development [40,41,42]. Therefore, the differentially expressed transcription factor identified between the IE and RE regions here might have different functions in grain protein deposition. Two transcription factors, (bZIP, TraesCS7B01G391800, and GRAS, TraesCS7D01G217500) were identified both in HN-IE vs. HN-RE and LN-IE vs. LN-RE comparisons, and the gene functional validation by the BMSV-VIGS experiment also confirmed that they are related to GSP content accumulation. Furthermore, close correlations were also observed between the gene encoding the bZIP transcription factor (TraesCS7B01G391800) and the metabolites dopamine, glutamine, and phenylalanine. What needs to be mentioned is that dopamine is mainly found in animals. However, it is also detected in many plants, and its biosynthetic pathway is similar to that in animals [43,44]. Ciepiela and Sempruch [45] found that the resistance of winter wheat to grain aphids was highly correlated with the concentration of levodopa, the natural precursor of the dopamine. Here, the differentially expressed dopamine between IR and RE may be attributed to different metabolic processes in the IE and RE regions. The study on potato starchy tubers showed that the content of dopamine in potato tubers stored at room temperature presented no decrease with storage time [43], implying that the relationship of dopamine with carbohydrate synthesis and metabolism in plants is not consistent with that in mammals. Of course, we noticed that the relative abundance of tentatively identified dopamine in this study is very low. The underlying regulation mechanism of the transcription factor and the role of differentially expressed metabolites under nitrogen treatment require further study.

Nitrogen fertilization plays an important role in wheat yield and grain quality. Compared with the LN treatment, increasing the nitrogen application level activates metabolic processes [24] and enhances the accumulation of gluten macropolymer by facilitating the SUMOylation of PPIase [16]. Here, HN treatment upregulated many genes, especially in the RE region. However, the upregulation of genes related to proteolysis mainly included those encoding serine-type endopeptidases, cysteine peptidases, and corresponding endopeptidase inhibitors, which indicates that complex hydrolysis processes regulate protein synthesis. Zhen et al. [24] proposed that accelerating the transformation between amino acids induced by HN treatment might promote GSP synthesis. It was reported that asparagine, glutamine, and glutamate are crucial for N translocation and storage [46] and that the abundance of these N-compounds might increase grain protein content [24]. Here, we also found that asparagine and glutamine accumulation was induced by HN treatment, which is consistent with the increased protein content observed in the HN treatment. The HN treatment caused more DEGs in the RE region than in the IE region, including transcripts involved in nitrogen metabolism, protein modification, and those encoding transcription factors. Moreover, the contents of amino acids and N-compounds were upregulated in the outer endosperm region in response to HN treatment. However, all the carbon metabolites detected in this study were highly abundant under HN treatment, irrespective of the endosperm region type. These results possibly indicate that high nitrogen availability contributes more to grain yield than increasing the GSP content, especially in the innermost endosperm region. We propose that HN activates metabolism in the outer endosperm region. In addition, the expression of 10 transcription factor genes was upregulated upon HN treatment in both endosperm regions. These transcription factors might be important for the response to nitrogen fertilizer application; further study of the regulatory target proteins and their functions will identify key genes related to nitrogen use efficiency.

## 4. Materials and Methods

### 4.1. Experimental Design and Plant Material

A winter wheat (*Triticum aestivum* L.) cultivar ‘Zhoumai36′ (this cultivar was bred by Henan, China, and the seed was provided by the breeder) was planted during the 2017–2018 growing season at the Xuchang experimental station of the National Engineering Research Center for Wheat, Henan province, China (34°08′N, 114°02′E). The soil is loamy Fluvoaquic, containing organic material (15.6 g kg^−1^, 0–30 cm), available phosphorus (37.5 mg kg^−1^), available potassium (119.0 mg kg^−1^), hydrolysable nitrogen (115 mg kg^−1^), and total nitrogen (1.1 g kg^−1^). Two nitrogen fertilization treatments with three replicates each were applied as follows: LN (0 kg ha^−1^) or HN (210 kg ha^−1^). Each plot received 0.299 kg K_2_O and 0.299 kg P_2_O_5_ before sowing. Half (50%) of the total nitrogen fertilizer (urea) was supplied before sowing, and another 50% was top-dressed at the jointing stage. Seeds were sown on 16 October 2017 at a density of 247 seed m^−2^. The plot dimensions were 4 × 5 m and field trials were managed according to local agronomic practices.

### 4.2. Sampling

**Figure 8:**
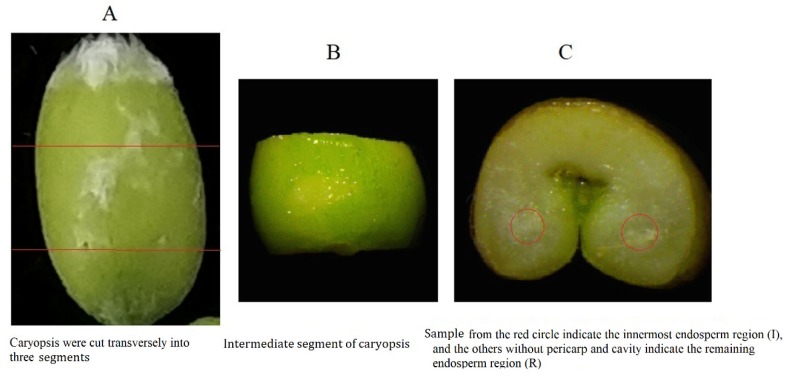
Division of different regions of wheat grain endosperm. (**A**) The red line indicate the transection location. (**B**) The intermediate segment of caryopsis is divided into the innermost endosperm region and the remaining endosperm region. (**C**) The red circle indicated the innermost endosperm region.

At the wheat flowering stage, spikes of a similar size that were undergoing anthesis on the same day were tagged. Grains at the first and second floret position at the center of each spike of wheat caryopses were collected 25 days after anthesis (DAA). Each grain was cut transversely into three sections (Figure 8A). After removing the pericarp, the central section was then divided into two parts under a stereomicroscope (Figure 8B,C). According to Savill et al. [5], the endosperm can be divided into five different tissues from the outer to inner parts. Here, the central two lobe zones of the grain were collected to represent the innermost endosperm (IE) region, and the remainder of the endosperm was collected as the remaining endosperm (RE) region. In this study, RE is a mix of tissues, including the living aleurone and the starchy endosperm. At maturity, plants in a 6-m^2^ area in each plot were harvested and grain yield was determined by weighing the harvested seeds.

### 4.3. Transcript Profiling

Total RNA of wheat endosperm was extracted from three biological replicates using a SpectrumTM Plant Total RNA Kit (Sigma, St Louis,, MO, USA), following the manufacturer’s instructions. RNA quantity and quality were evaluated using a Nanodrop spectrophotometer and Agilent 2100 RNA Nano 6000 Assay Kit (Agilent Technologies, Palo Alto, CA, USA). In total, 12 independent endosperm cDNA libraries were constructed according to Illumina’s TruSequ RNA sample preparation. The quality of cDNA libraries was assessed by testing the insert size using Agilent 2100, and a library effective concentration >2 nM was used for sequencing by an Illumina HiSeq 2500 System (Illumina, Foster, CA, USA). The raw sequencing reads were referred to the Illumina pipeline filter (Solexa 0.3), and then an in-house program (Fastp, https://github.com/OpenGene/fastp) was used to process the dataset for removing adapter dimers, poly-N, and low-quality reads. The clean reads were mapped onto the wheat “Chinese Spring” reference genome (IWGSC1_popseq.31) using HISAT2 [47]. The raw sequence data has been deposited in a Gene Expression Omnibus (GEO) repository with accession numbers GSE133846 (https://www.ncbi.nlm.nih.gov/geo/query/acc.cgi?acc=GSE133846).

Gene expression levels were identified and normalized using fragments per kilobase of transcript sequence per millions base pairs sequenced (FPKM) [48]. The differentially expressed genes between different treatments were calculated using DEGseq2 [49], and a *p*-value ≤0.05, and |log_2_fold change|≥1 were defined as thresholds. To predict the functions of the identified genes, BLASTx searches were performed against Gene Ontology (http://www.geneontology.org/), Kyoto encyclopedia of genes and genomes (KEGG, http://www.genome.jp/kegg/), STRING (http://string-db.org/), UniProt/Swiss-Prot (https://www.uniprot.org), and rMATS (http://rnaseq-mats.sourceforge.net/index.html) databases.

### 4.4. Gene Expression Quantification Using qPCR

Reverse-transcription was carried out using the RNA First-strand cDNA Synthesis SuperMix (TransScript), according to the manufacturer′s instructions. A SYBR PrimeScript miRNA RT-PCR Kit was used to perform qPCR reactions on a CFX96TM Real-Time System (C1000TM Thermal cycler, BIO-RAD, Foster, CA, USA). The relative expression levels were calculated using the 2^−∆∆*C*t^ method [50]. The transcript-specific primer sequences used in this study are provided in Supplementary Table S4.

### 4.5. Grain Metabolite Extraction

Six biological replicate grain samples from each treatment were ground to homogeneity in liquid nitrogen. After vacuum freeze-drying, 60 mg powder was homogenized in 1.6 mL extraction buffer (methanol: Acetonitrile: Water, 2:2:1, *v*/*v*). The samples were mixed thoroughly and subjected to ultrasonic treatment for 15 min and stored for 1 h at −20 ℃. The mixtures were then centrifuged for 15 min at 13,000 rpm, and the supernatant was collected and dried under vacuum.

### 4.6. Metabolomic Analysis

Samples were separated using ultra high performance liquid chromatography (UHPLC) (Agilent 1290 Infinity LC, Agilent Technologies), and tandem mass spectrometry analyses were performed with a Triple TOF5600 (AB SCIEX). To reduce system errors, samples were analyzed in a random order. The (electro spray ionization (ESI) source conditions were: Ion Source Gas1(Gas), 40; Ion Source Gas2(Gas2), 60; curtain gas (CUR), 30; source temperature, 600 °C; IonSapary Voltage Floating (ISVF) ± 5500 V (positive and negative modes); a time of flight mass spectrometry (TOF MS) scan m/z range, 60–1,000 Da; a product ion scan *m*/*z* range, 25–1,000 Da; TOF MS scan accumulation time 0.20 s/spectra, product ion scan accumulation time 0.05 s/spectra.

The raw data were preprocessed in mzXML format using XCMS (https://xcmsonline.scripps.edu./) for retention time correction, chromatogram alignment, and peak area extraction. The processed data were subjected to multi-dimensional statistical analysis, including unsupervised principal component analysis (PCA), supervised partial least squares-discriminant analysis (PLS-DA), and orthogonal partial least squares discriminant analysis (OPLS-DA), after processing by Pareto-scaling using SIMCA-P 14.1 (Umetric, Umea, Sweden). Metabolites were tentatively identified by matching the data to the NIST database (http://www.nist.gov/srd/nist1a.html) and Wiley 9 database (http://www.sisweb.com/software/ms/wiley/hyml). Differentially expressed metabolites between different treatments were evaluated using variable importance for the projection (VIP) from the OPLS-DA model. VIP scores were used to estimate the importance of each variable in the projection, and VIP >1 was often used as a variable selection criterion. A *p*-value ≤0.05 and VIP >1 defined significantly differential metabolites and a 0.05≥ *p*-value ≤0.1 and VIP >1 defined differentially abundant metabolites.

### 4.7. Functional Gene Analysis Using the Barley Stripe Mosaic Virus (BSMV)-Based Virus-Induced Gene Silencing (VIGS) System

The BSMV-VIGS system is an effective and convenient technology to perform functional gene analysis. Two genes (TraesCS7B01G391800 and TraesCS2B01G217500, named *TabZIP* and *TaGRAS,* respectively) were functionally analyzed using the BSMV-VIGS approach. BSMV:00 with no insert was used as an empty vector control. The sequences of *TabZIP* and *TaGRAS* from wheat cultivar “Zhoumai36” were obtained by polymerase chain reaction (PCR) amplification, according to the sequences of TraesCS7B01G391800 and TraesCS2B01G217500. The BSMV construct carrying a 226-bp fragment of *TabZIP* from the gene-coding region (136 bp to 361 bp downstream of the start codon) was generated and used to silence the *bZIP* gene, named BSMV:bZIP. The fragment containing the 97-bp fragment of *TaGRAS* from the gene coding region (818 bp to 914 bp downstream from the start codon) was generated as BSMV:GRAS. At the heading stage, thirty spikes were infected with each recombinant vector following the method of Ma et al. [51].

### 4.8. Determination of Grain Total Protein Content and the Individual Fraction Contents

Wheat-grain protein fractions, including albumins, globulins, gliadins, and glutenins, were extracted according to the method of Liu et al. [52]. Protein concentrations were determined using a Kjeldahl apparatus (Kjeltec 2300, FOSS, Hoganas, Sweden), according to the manufacturer’s instructions.

## 5. Conclusions

In summary, this study demonstrates that the protein content in the RE region had a greater response to the supplied nitrogen than the IE region. Several candidate genes involved in carbon and nitrogen metabolism, and those encoding transcription factors and proteins involved in post-translation modification potentially related to the difference between the IR and RE regions, and their responses to nitrogen supplying were identified. These DEGs might play important functions by increasing the supply and transport of amino acids. Our results provide novel insights into the molecular mechanism underlying the differences in GSP content between the IE and RE regions and its response to nitrogen fertilizer supply.

## Figures and Tables

**Table 1:** Protein content of the innermost endosperm (IE) region and the remaining endosperm (RE) region from plants grown in high nitrogen (HN) and low nitrogen (LN) treatments.

Item.		Total Protein Content (mg g^−1^)	Albumin Content (mg g^−1^)	Globulin Content (mg g^−1^)	Gliadin Content (mg g^−1^)	Glutenin Content (mg g^−1^)	Grain Yield (kg ha^−1^)
HN	IE	118.5 ± 2.1 ^b^	32.0 ± 3.0 ^b^	11.5 ± 1.6 ^b^^,^^c^	23.1 ± 0.1 ^c^	46.1 ± 0.4 ^a,b^	7241.5 ± 84.6 ^a^
	RE	137.5 ± 0.7 ^a^	36.9 ± 1.6 ^a^	19.4 ± 1.1 ^a^	25.6 ± 0.1 ^b^	49.0 ± 3.3 ^a^	
LN	IE	101.5 ± 2.4 ^c^	29.6 ± 0.5 ^b^	8.4 ± 2.1 ^c^	19.9 ± 0.6 ^d^	43.0 ± 1.0 ^b^	5864.6 ± 131 ^b^
	RE	116.5 ± 1.2 ^b^	30.8 ± 2.4 ^b^	12.3 ± 0.8 ^b^	26.5 ± 1.3 ^a^	44.8 ± 1.4 ^a,b^	

^a^ Values expressed as mean ± standard deviation. ^b^ Within a column, mean values followed by different lowercase letters are significantly different at the *p* < 0.05 (least significant difference). ^c^ HN and LN represent the high nitrogen and low nitrogen fertilizer application level, respectively. ^d^ IE and RE represent for the innermost endosperm region and the remaining endosperm region, respectively.

## Supplementary Materials

Supplementary materials can be found at https://www.mdpi.com/1422-0067/20/17/4212/s1.
