# Supplementary material for: Transcriptomic and Metabolomics Analysis of Different Endosperm Region under Nitrogen Treatments
Source: Int J Mol Sci. 2019 Aug 28;20(17):4212. doi: 10.3390/ijms20174212 (PMC6747615; doi:10.3390/ijms20174212)
Supplement: Supplementary file 1 [file ijms-20-04212-s001.zip › ijms-575327-for final-suppl/Supplementary-Table S1.docx]

**Table S1-I** Differentially expressed genes between the inner and outer endosperm under both HN and LN treatment.

| **Gene-ID** | **LN-I**  **Readcount** | **LN-O**  **Readcount** | **log2**  **Foldchange** | ***p-*Value** | **Padj** | **HN-I**  **Readcount** | **HN-O**  **Readcount** | **log2**  **Foldchange** | ***p-*Value** | **Padj** |
| --- | --- | --- | --- | --- | --- | --- | --- | --- | --- | --- |
| Novel.16625 | 98.53 | 32.33 | 1.62 | 0.00 | 0.01 | 78.07 | 12.11 | 2.69 | 0.00 | 0.00 |
| Novel.38988 | 2048.71 | 689.66 | 1.57 | 0.00 | 0.01 | 4619.22 | 1435.05 | 1.69 | 0.00 | 0.00 |
| TraesCS1A01G191800 | 163.23 | 47.78 | 1.77 | 0.00 | 0.02 | 335.64 | 130.94 | 1.36 | 0.00 | 0.00 |
| TraesCS1D01G059500 | 74.17 | 9.34 | 2.99 | 0.00 | 0.00 | 174.39 | 44.33 | 1.97 | 0.00 | 0.00 |
| TraesCS1D01G101900 | 312.5 | 42.15 | 2.89 | 0.00 | 0.00 | 1157.61 | 338.56 | 1.77 | 0.00 | 0.00 |
| TraesCS1D01G320100 | 357.4 | 146.54 | 1.28 | 0.00 | 0.04 | 419.76 | 196.92 | 1.09 | 0.00 | 0.00 |
| TraesCS2B01G131200 | 164.34 | 47.14 | 1.79 | 0.00 | 0.00 | 1030.47 | 509.05 | 1.02 | 0.00 | 0.02 |
| TraesCS2B01G217500 | 176.07 | 39.89 | 2.14 | 0.00 | 0.00 | 395.53 | 124.02 | 1.67 | 0.00 | 0.00 |
| TraesCS2B01G359400 | 400.49 | 107.83 | 1.89 | 0.00 | 0.01 | 1274.17 | 550.72 | 1.21 | 0.00 | 0.01 |
| TraesCS2B01G546700 | 390.23 | 58.85 | 2.73 | 0.00 | 0.00 | 759.12 | 163.47 | 2.21 | 0.00 | 0.00 |
| TraesCS3A01G181400 | 115.93 | 30.47 | 1.92 | 0.00 | 0.02 | 256.9 | 82.79 | 1.63 | 0.00 | 0.00 |
| TraesCS3A01G303300 | 1362.62 | 591.9 | 1.20 | 0.00 | 0.01 | 1992.23 | 881.71 | 1.18 | 0.00 | 0.02 |
| TraesCS3B01G211400 | 83.91 | 18.68 | 2.14 | 0.00 | 0.00 | 248.36 | 86.7 | 1.51 | 0.00 | 0.00 |
| TraesCS3B01G515100 | 12139.8 | 3184.88 | 1.93 | 0.00 | 0.00 | 12357.5 | 2894.58 | 2.09 | 0.00 | 0.00 |
| TraesCS3D01G300100 | 124.29 | 41.75 | 1.57 | 0.00 | 0.04 | 183.47 | 55.06 | 1.74 | 0.00 | 0.01 |
| TraesCS4A01G200300 | 72.38 | 22.66 | 1.67 | 0.00 | 0.02 | 148.77 | 60.92 | 1.29 | 0.00 | 0.01 |
| TraesCS4D01G076000 | 329.55 | 104.04 | 1.66 | 0.00 | 0.00 | 243.56 | 94.07 | 1.37 | 0.00 | 0.01 |
| TraesCS4D01G124700 | 116.55 | 35.33 | 1.72 | 0.00 | 0.05 | 88.82 | 28.67 | 1.63 | 0.00 | 0.01 |
| TraesCS5A01G248900 | 74.89 | 17.11 | 2.12 | 0.00 | 0.01 | 35.16 | 7.05 | 2.32 | 0.00 | 0.02 |
| TraesCS5B01G021000 | 102.84 | 24.1 | 2.10 | 0.00 | 0.00 | 75.09 | 12.44 | 2.62 | 0.00 | 0.00 |
| TraesCS5B01G064000 | 79.15 | 19.46 | 2.01 | 0.00 | 0.01 | 192.13 | 45 | 2.10 | 0.00 | 0.00 |
| TraesCS5B01G219500 | 73.38 | 15.29 | 2.26 | 0.00 | 0.02 | 107.38 | 27.81 | 1.95 | 0.00 | 0.01 |
| TraesCS5D01G033100 | 111.41 | 41.83 | 1.40 | 0.00 | 0.04 | 171.01 | 57.08 | 1.58 | 0.00 | 0.00 |
| TraesCS5D01G199500 | 326.61 | 62.04 | 2.40 | 0.00 | 0.04 | 1728.39 | 560.87 | 1.62 | 0.00 | 0.00 |
| TraesCS5D01G380600 | 57.56 | 16.95 | 1.76 | 0.00 | 0.03 | 342.37 | 125.76 | 1.44 | 0.00 | 0.01 |
| TraesCS5D01G400800 | 633.17 | 144.35 | 2.13 | 0.00 | 0.05 | 451.37 | 150.8 | 1.58 | 0.00 | 0.02 |
| TraesCS5D01G408100 | 213.57 | 40.96 | 2.38 | 0.00 | 0.02 | 903.83 | 125.72 | 2.85 | 0.00 | 0.00 |
| TraesCS7A01G488600 | 786.48 | 168.65 | 2.22 | 0.00 | 0.00 | 673.88 | 185 | 1.86 | 0.00 | 0.01 |
| TraesCS7B01G391800 | 830.37 | 227.37 | 1.87 | 0.00 | 0.01 | 706.02 | 249.85 | 1.50 | 0.00 | 0.03 |
| TraesCS7D01G512700 | 707.4 | 236.75 | 1.58 | 0.00 | 0.00 | 306.56 | 84.82 | 1.85 | 0.00 | 0.00 |
| Novel.13153 | 17.46 | 158.91 | -3.14 | 0.00 | 0.00 | 34.06 | 134.28 | -1.98 | 0.00 | 0.03 |
| Novel.15335 | 101.93 | 331.46 | -1.70 | 0.00 | 0.00 | 128.67 | 289.08 | -1.17 | 0.00 | 0.01 |
| Novel.16013 | 64.09 | 324.66 | -2.32 | 0.00 | 0.00 | 117.07 | 278.05 | -1.25 | 0.00 | 0.01 |
| Novel.19583 | 96.54 | 282.18 | -1.54 | 0.00 | 0.01 | 134 | 289.23 | -1.11 | 0.00 | 0.01 |
| Novel.22240 | 61.07 | 407.37 | -2.72 | 0.00 | 0.01 | 91.38 | 197.79 | -1.11 | 0.00 | 0.02 |
| Novel.22368 | 6.07 | 40.71 | -2.67 | 0.00 | 0.00 | 9.22 | 68.32 | -2.89 | 0.00 | 0.01 |
| Novel.2327 | 102.63 | 421.01 | -2.03 | 0.00 | 0.00 | 88 | 236.78 | -1.43 | 0.00 | 0.01 |
| Novel.35331 | 24.12 | 82.4 | -1.75 | 0.00 | 0.03 | 24.94 | 83.19 | -1.73 | 0.00 | 0.01 |
| Novel.36401 | 15.58 | 67.95 | -2.07 | 0.00 | 0.01 | 17.41 | 58.72 | -1.74 | 0.00 | 0.03 |
| Novel.36648 | 26.51 | 214.9 | -2.98 | 0.00 | 0.00 | 45.89 | 110.14 | -1.26 | 0.00 | 0.05 |
| Novel.38644 | 39.47 | 224.59 | -2.49 | 0.00 | 0.02 | 67.46 | 169.61 | -1.33 | 0.00 | 0.01 |
| Novel.40220 | 19.91 | 127.9 | -2.64 | 0.00 | 0.00 | 48.04 | 143.74 | -1.58 | 0.00 | 0.00 |
| Novel.4309 | 38.5 | 192.15 | -2.29 | 0.00 | 0.04 | 73.34 | 174.23 | -1.25 | 0.00 | 0.03 |
| Novel.4338 | 90.32 | 415.44 | -2.19 | 0.00 | 0.00 | 131.28 | 301.06 | -1.20 | 0.00 | 0.03 |
| Novel.48954 | 19.1 | 160.06 | -3.03 | 0.00 | 0.00 | 44.55 | 125.73 | -1.50 | 0.00 | 0.00 |
| Novel.51300 | 513.31 | 1211.74 | -1.24 | 0.00 | 0.00 | 361.65 | 809.58 | -1.16 | 0.00 | 0.02 |
| Novel.51363 | 12.78 | 143.7 | -3.43 | 0.00 | 0.00 | 27.45 | 94.12 | -1.78 | 0.00 | 0.01 |
| Novel.57788 | 21.81 | 208.35 | -3.22 | 0.00 | 0.00 | 71.31 | 231.42 | -1.70 | 0.00 | 0.00 |
| Novel.58091 | 96.07 | 605.52 | -2.65 | 0.00 | 0.00 | 117.56 | 287.22 | -1.29 | 0.00 | 0.03 |
| Novel.7229 | 22.17 | 110.07 | -2.31 | 0.00 | 0.00 | 20.9 | 72.01 | -1.79 | 0.00 | 0.01 |
| Novel.7578 | 30.95 | 124.16 | -1.99 | 0.00 | 0.02 | 30.83 | 100.49 | -1.71 | 0.00 | 0.01 |
| Novel.9669 | 30.6 | 182.39 | -2.55 | 0.00 | 0.00 | 41.53 | 103.12 | -1.32 | 0.00 | 0.02 |
| TraesCS1A01G150200 | 92.36 | 359.16 | -1.95 | 0.00 | 0.01 | 122.47 | 282.27 | -1.20 | 0.00 | 0.00 |
| TraesCS1A01G406300 | 121.45 | 485.97 | -1.99 | 0.00 | 0.01 | 330.41 | 978.47 | -1.57 | 0.00 | 0.00 |
| TraesCS1B01G183100 | 2573.51 | 8324.48 | -1.69 | 0.00 | 0.03 | 3823.18 | 11817.39 | -1.63 | 0.00 | 0.00 |
| TraesCS1B01G445400 | 107.48 | 513.64 | -2.25 | 0.00 | 0.00 | 131.46 | 311.47 | -1.24 | 0.00 | 0.00 |
| TraesCS1D01G012000 | 28.73 | 196.44 | -2.74 | 0.00 | 0.00 | 124.16 | 410.01 | -1.72 | 0.00 | 0.00 |
| TraesCS1D01G040600 | 149.67 | 495.44 | -1.72 | 0.00 | 0.04 | 165.02 | 424.09 | -1.36 | 0.00 | 0.03 |
| TraesCS1D01G163800 | 394.42 | 1712.3 | -2.11 | 0.00 | 0.02 | 631.29 | 1735.82 | -1.46 | 0.00 | 0.00 |
| TraesCS1D01G421200 | 167.28 | 521.4 | -1.64 | 0.00 | 0.00 | 88.46 | 218.21 | -1.30 | 0.00 | 0.02 |
| TraesCS1D01G422900 | 255.35 | 1252.82 | -2.29 | 0.00 | 0.00 | 416.59 | 936.75 | -1.17 | 0.00 | 0.02 |
| TraesCS2A01G522100 | 83.27 | 322.57 | -1.94 | 0.00 | 0.01 | 321.16 | 724.06 | -1.17 | 0.00 | 0.01 |
| TraesCS2B01G266000 | 160.64 | 418.61 | -1.37 | 0.00 | 0.03 | 174.83 | 363.54 | -1.06 | 0.00 | 0.04 |
| TraesCS2D01G109600 | 34.46 | 163.47 | -2.22 | 0.00 | 0.04 | 25.7 | 96.6 | -1.91 | 0.00 | 0.04 |
| TraesCS2D01G402300 | 117.82 | 437.01 | -1.88 | 0.00 | 0.02 | 107.22 | 310.83 | -1.54 | 0.00 | 0.02 |
| TraesCS2D01G536300 | 408.29 | 1351.36 | -1.72 | 0.00 | 0.05 | 252.77 | 912.01 | -1.85 | 0.00 | 0.01 |
| TraesCS3A01G103800 | 82.27 | 313.54 | -1.92 | 0.00 | 0.00 | 97.87 | 232.6 | -1.25 | 0.00 | 0.00 |
| TraesCS3A01G140800 | 578.01 | 1990.32 | -1.78 | 0.00 | 0.02 | 706.79 | 1724.17 | -1.29 | 0.00 | 0.01 |
| TraesCS3A01G202800 | 36.85 | 138.11 | -1.89 | 0.00 | 0.00 | 62.56 | 166.46 | -1.41 | 0.00 | 0.04 |
| TraesCS3A01G454700 | 522.55 | 1957.16 | -1.90 | 0.00 | 0.04 | 489.5 | 1497.02 | -1.61 | 0.00 | 0.03 |
| TraesCS3B01G012300 | 24.61 | 104.76 | -2.05 | 0.00 | 0.04 | 23.63 | 89.81 | -1.92 | 0.00 | 0.05 |
| TraesCS3B01G560100 | 49.59 | 210.96 | -2.07 | 0.00 | 0.00 | 24.31 | 118.32 | -2.27 | 0.00 | 0.00 |
| TraesCS3D01G206400 | 61.1 | 142.54 | -1.21 | 0.00 | 0.04 | 65.7 | 164.11 | -1.32 | 0.00 | 0.04 |
| TraesCS3D01G447500 | 523.7 | 1937.77 | -1.89 | 0.00 | 0.04 | 487.9 | 1530.23 | -1.65 | 0.00 | 0.03 |
| TraesCS3D01G506400 | 637.83 | 1962.75 | -1.62 | 0.00 | 0.02 | 805.46 | 1838.31 | -1.19 | 0.00 | 0.01 |
| TraesCS4A01G052100 | 173.73 | 657.32 | -1.91 | 0.00 | 0.02 | 184.27 | 619.53 | -1.75 | 0.00 | 0.01 |
| TraesCS4A01G075100 | 195.03 | 590.62 | -1.59 | 0.00 | 0.02 | 165.68 | 388.49 | -1.23 | 0.00 | 0.01 |
| TraesCS4A01G369300 | 29.14 | 103.44 | -1.79 | 0.00 | 0.05 | 63.3 | 161.11 | -1.35 | 0.00 | 0.03 |
| TraesCS4A01G386000 | 62.84 | 255.39 | -2.01 | 0.00 | 0.01 | 72.59 | 256.8 | -1.82 | 0.00 | 0.04 |
| TraesCS4A01G386700 | 40.46 | 176.72 | -2.10 | 0.00 | 0.01 | 83.15 | 199.28 | -1.26 | 0.00 | 0.03 |
| TraesCS4A01G460500 | 39.14 | 178.71 | -2.16 | 0.00 | 0.00 | 63.76 | 228.54 | -1.84 | 0.00 | 0.00 |
| TraesCS4A01G460600 | 26.69 | 111.76 | -2.03 | 0.00 | 0.00 | 46.12 | 146.35 | -1.67 | 0.00 | 0.05 |
| TraesCS4B01G277000 | 23.12 | 87.5 | -1.90 | 0.00 | 0.04 | 12.74 | 66.72 | -2.40 | 0.00 | 0.02 |
| TraesCS4B01G344300 | 226.58 | 680.44 | -1.58 | 0.00 | 0.03 | 244.86 | 508.31 | -1.05 | 0.00 | 0.02 |
| TraesCS4B01G344700 | 1230.13 | 3461.1 | -1.49 | 0.00 | 0.01 | 1477.99 | 3156.09 | -1.09 | 0.00 | 0.00 |
| TraesCS4D01G275600 | 199.51 | 645.18 | -1.69 | 0.00 | 0.04 | 108.83 | 413.78 | -1.93 | 0.00 | 0.01 |
| TraesCS4D01G298300 | 10.52 | 64.32 | -2.54 | 0.00 | 0.00 | 21.45 | 106.09 | -2.30 | 0.00 | 0.00 |
| TraesCS4D01G339400 | 258.18 | 1223.96 | -2.24 | 0.00 | 0.00 | 451.95 | 1000.24 | -1.15 | 0.00 | 0.00 |
| TraesCS5A01G283100 | 1244.14 | 4601.01 | -1.89 | 0.00 | 0.00 | 1244.14 | 4601.01 | -1.89 | 0.00 | 0.00 |
| TraesCS5A01G369800 | 31.77 | 191.97 | -2.57 | 0.00 | 0.00 | 49.63 | 217.52 | -2.12 | 0.00 | 0.00 |
| TraesCS5B01G282100 | 3326.8 | 11427.07 | -1.78 | 0.00 | 0.02 | 2712.3 | 10823.95 | -2.00 | 0.00 | 0.00 |
| TraesCS5B01G400600 | 3850.77 | 12578.5 | -1.71 | 0.00 | 0.03 | 3775.87 | 9631.53 | -1.35 | 0.00 | 0.01 |
| TraesCS5B01G424800 | 2291.15 | 7200.77 | -1.65 | 0.00 | 0.04 | 1986.8 | 6094.89 | -1.62 | 0.00 | 0.02 |
| TraesCS5B01G491800 | 9.29 | 51.96 | -2.38 | 0.00 | 0.05 | 10.37 | 45.72 | -2.12 | 0.00 | 0.04 |
| TraesCS5B01G566500 | 186.7 | 558.86 | -1.57 | 0.00 | 0.03 | 266.96 | 581.66 | -1.12 | 0.00 | 0.02 |
| TraesCS5D01G379200 | 313.41 | 1044.39 | -1.73 | 0.00 | 0.03 | 371.64 | 1152.78 | -1.63 | 0.00 | 0.00 |
| TraesCS5D01G431100 | 3387.72 | 11023.49 | -1.70 | 0.00 | 0.04 | 3053.44 | 9336.3 | -1.61 | 0.00 | 0.02 |
| TraesCS5D01G492300 | 25.08 | 151.93 | -2.56 | 0.00 | 0.00 | 39.31 | 158.53 | -2.01 | 0.00 | 0.00 |
| TraesCS6A01G017900 | 238.1 | 948.86 | -1.99 | 0.00 | 0.03 | 265.85 | 683.98 | -1.36 | 0.00 | 0.04 |
| TraesCS6B01G118000 | 7.86 | 64.89 | -2.97 | 0.00 | 0.00 | 4.25 | 28.39 | -2.75 | 0.00 | 0.03 |
| TraesCS6D01G138200 | 642.89 | 2234.01 | -1.80 | 0.00 | 0.04 | 296.59 | 1238.44 | -2.06 | 0.00 | 0.00 |
| TraesCS6D01G159200 | 29.26 | 127.64 | -2.10 | 0.00 | 0.03 | 26.04 | 110.33 | -2.08 | 0.00 | 0.01 |
| TraesCS7A01G087800 | 20.59 | 111.49 | -2.39 | 0.00 | 0.03 | 24.58 | 77.72 | -1.68 | 0.00 | 0.02 |
| TraesCS7D01G448200 | 64.53 | 201.73 | -1.63 | 0.00 | 0.03 | 42.55 | 158.49 | -1.90 | 0.00 | 0.03 |

**Table S1-II** Differentially expressed genes between HN and LN treatment in both inner and outer endosperm.

| **Gene-ID** | **LN-I**  **Readcount** | **HN-I**  **Readcount** | **log2**  **FoldChange** | ***p*-Value** | **Padj** | **LN-O**  **Readcount** | **HN-O**  **Readcount** | **log2**  **FoldChange** | ***p*-Value** | **Padj** |
| --- | --- | --- | --- | --- | --- | --- | --- | --- | --- | --- |
| Novel.10609 | 161.94 | 65.93 | 1.29 | 0.00 | 0.05 | 174.55 | 81.87 | 1.10 | 0.00 | 0.03 |
| Novel.13419 | 227.35 | 59.56 | 1.92 | 0.00 | 0.00 | 169.44 | 68.45 | 1.31 | 0.00 | 0.00 |
| Novel.14055 | 1388.90 | 481.74 | 1.53 | 0.00 | 0.01 | 1152.66 | 499.82 | 1.21 | 0.00 | 0.01 |
| Novel.16143 | 69.98 | 20.33 | 1.78 | 0.00 | 0.00 | 59.21 | 19.44 | 1.61 | 0.00 | 0.01 |
| Novel.17326 | 123.34 | 25.80 | 2.26 | 0.00 | 0.00 | 134.82 | 34.03 | 1.98 | 0.00 | 0.00 |
| Novel.24973 | 322.68 | 105.80 | 1.61 | 0.00 | 0.04 | 412.28 | 161.80 | 1.35 | 0.00 | 0.00 |
| Novel.33492 | 267.04 | 108.71 | 1.29 | 0.00 | 0.00 | 292.53 | 131.68 | 1.15 | 0.00 | 0.00 |
| Novel.34936 | 144.64 | 45.82 | 1.65 | 0.00 | 0.01 | 149.71 | 49.43 | 1.60 | 0.00 | 0.00 |
| Novel.39932 | 109.01 | 44.93 | 1.27 | 0.00 | 0.04 | 131.84 | 56.52 | 1.23 | 0.00 | 0.01 |
| Novel.42379 | 388.47 | 187.01 | 1.06 | 0.00 | 0.04 | 541.87 | 239.01 | 1.18 | 0.00 | 0.00 |
| Novel.45566 | 2600.05 | 1153.89 | 1.17 | 0.00 | 0.03 | 3311.98 | 1302.27 | 1.35 | 0.00 | 0.01 |
| Novel.47024 | 80.68 | 15.32 | 2.38 | 0.00 | 0.00 | 68.27 | 20.05 | 1.77 | 0.00 | 0.02 |
| Novel.47170 | 216.43 | 73.11 | 1.57 | 0.00 | 0.00 | 203.73 | 81.40 | 1.32 | 0.00 | 0.01 |
| Novel.55111 | 1042.87 | 354.65 | 1.55 | 0.00 | 0.00 | 1079.74 | 501.84 | 1.10 | 0.00 | 0.02 |
| Novel.56033 | 259.97 | 96.54 | 1.43 | 0.00 | 0.02 | 156.30 | 54.09 | 1.53 | 0.00 | 0.00 |
| Novel.58188 | 239.61 | 58.71 | 2.03 | 0.00 | 0.00 | 303.23 | 82.32 | 1.89 | 0.00 | 0.00 |
| TraesCS1A01G215300 | 307.34 | 140.59 | 1.13 | 0.00 | 0.01 | 424.27 | 118.94 | 1.83 | 0.00 | 0.00 |
| TraesCS1A01G337900 | 210.94 | 93.90 | 1.17 | 0.00 | 0.02 | 459.08 | 177.03 | 1.37 | 0.00 | 0.00 |
| TraesCS1A01G443000 | 787.64 | 355.94 | 1.15 | 0.00 | 0.03 | 821.92 | 405.99 | 1.02 | 0.00 | 0.05 |
| TraesCS1B01G477100 | 757.98 | 359.94 | 1.07 | 0.00 | 0.05 | 812.64 | 378.54 | 1.10 | 0.00 | 0.00 |
| TraesCS1D01G107700 | 163.88 | 40.76 | 2.00 | 0.00 | 0.00 | 114.94 | 42.93 | 1.43 | 0.00 | 0.01 |
| TraesCS2A01G014300 | 711.62 | 247.94 | 1.52 | 0.00 | 0.00 | 855.79 | 238.13 | 1.85 | 0.00 | 0.00 |
| TraesCS2A01G173200 | 866.32 | 379.67 | 1.19 | 0.00 | 0.02 | 877.77 | 380.98 | 1.20 | 0.00 | 0.00 |
| TraesCS2A01G184300 | 76.89 | 30.63 | 1.32 | 0.00 | 0.05 | 100.90 | 35.98 | 1.49 | 0.00 | 0.02 |
| TraesCS2A01G224100 | 258.06 | 93.02 | 1.48 | 0.00 | 0.04 | 240.93 | 90.02 | 1.42 | 0.00 | 0.01 |
| TraesCS2A01G386800 | 1287.15 | 364.92 | 1.82 | 0.00 | 0.01 | 1610.26 | 617.13 | 1.38 | 0.00 | 0.02 |
| TraesCS2A01G452000 | 83.99 | 25.39 | 1.72 | 0.00 | 0.01 | 176.04 | 68.98 | 1.35 | 0.00 | 0.04 |
| TraesCS2A01G455400 | 1538.10 | 767.17 | 1.00 | 0.00 | 0.01 | 1494.01 | 569.98 | 1.39 | 0.00 | 0.00 |
| TraesCS2A01G563100 | 297.91 | 118.10 | 1.33 | 0.00 | 0.00 | 235.96 | 84.95 | 1.48 | 0.00 | 0.00 |
| TraesCS2A01G577100 | 7289.01 | 2683.18 | 1.44 | 0.00 | 0.00 | 8412.08 | 3264.36 | 1.37 | 0.00 | 0.00 |
| TraesCS2A01G587900 | 567.34 | 119.73 | 2.24 | 0.00 | 0.00 | 298.53 | 49.89 | 2.59 | 0.00 | 0.00 |
| TraesCS2B01G002100 | 1551.30 | 486.09 | 1.67 | 0.00 | 0.00 | 1567.87 | 489.13 | 1.68 | 0.00 | 0.00 |
| TraesCS2B01G047400 | 2042.65 | 425.97 | 2.26 | 0.00 | 0.00 | 3197.35 | 1048.08 | 1.61 | 0.00 | 0.01 |
| TraesCS2B01G126700 | 68.79 | 20.35 | 1.78 | 0.00 | 0.05 | 105.80 | 33.96 | 1.65 | 0.00 | 0.00 |
| TraesCS2B01G223100 | 30.01 | 4.68 | 2.65 | 0.00 | 0.01 | 38.66 | 6.30 | 2.65 | 0.00 | 0.00 |
| TraesCS2B01G294500 | 1927.50 | 538.71 | 1.84 | 0.00 | 0.01 | 2297.05 | 672.28 | 1.77 | 0.00 | 0.00 |
| TraesCS2B01G540400 | 363.21 | 90.17 | 2.01 | 0.00 | 0.00 | 578.26 | 135.19 | 2.10 | 0.00 | 0.00 |
| TraesCS2B01G591100 | 130.30 | 44.25 | 1.55 | 0.00 | 0.03 | 240.58 | 69.01 | 1.81 | 0.00 | 0.00 |
| TraesCS2D01G015500 | 1368.56 | 447.28 | 1.61 | 0.00 | 0.00 | 1439.19 | 544.36 | 1.40 | 0.00 | 0.00 |
| TraesCS2D01G043700 | 618.38 | 201.95 | 1.62 | 0.00 | 0.00 | 499.88 | 223.93 | 1.16 | 0.00 | 0.01 |
| TraesCS2D01G158700 | 279.33 | 130.60 | 1.10 | 0.00 | 0.01 | 370.70 | 182.92 | 1.02 | 0.00 | 0.02 |
| TraesCS2D01G276000 | 2088.40 | 603.61 | 1.79 | 0.00 | 0.01 | 2310.11 | 810.38 | 1.51 | 0.00 | 0.02 |
| TraesCS2D01G383600 | 2452.04 | 792.63 | 1.63 | 0.00 | 0.02 | 2601.50 | 1203.97 | 1.11 | 0.00 | 0.04 |
| TraesCS2D01G552700 | 18615.49 | 8205.94 | 1.18 | 0.00 | 0.03 | 23382.06 | 8563.51 | 1.45 | 0.00 | 0.00 |
| TraesCS2D01G565300 | 316.28 | 114.79 | 1.46 | 0.00 | 0.01 | 141.50 | 34.45 | 2.04 | 0.00 | 0.00 |
| TraesCS2D01G572300 | 111.75 | 35.54 | 1.64 | 0.00 | 0.00 | 75.83 | 24.85 | 1.60 | 0.00 | 0.01 |
| TraesCS3A01G128800 | 89.54 | 17.28 | 2.38 | 0.00 | 0.00 | 91.50 | 37.60 | 1.28 | 0.00 | 0.02 |
| TraesCS3A01G149900 | 181.15 | 46.63 | 1.95 | 0.00 | 0.00 | 342.46 | 103.96 | 1.72 | 0.00 | 0.00 |
| TraesCS3A01G294500 | 340.03 | 133.80 | 1.34 | 0.00 | 0.02 | 495.98 | 223.62 | 1.15 | 0.00 | 0.05 |
| TraesCS3A01G357900 | 3374.07 | 1098.45 | 1.62 | 0.00 | 0.00 | 3887.85 | 1516.71 | 1.36 | 0.00 | 0.00 |
| TraesCS3A01G390000 | 12909.87 | 5282.19 | 1.29 | 0.00 | 0.01 | 17204.03 | 5349.35 | 1.69 | 0.00 | 0.00 |
| TraesCS3A01G399800 | 363.42 | 148.73 | 1.29 | 0.00 | 0.00 | 764.44 | 262.35 | 1.54 | 0.00 | 0.00 |
| TraesCS3B01G177000 | 73.54 | 16.58 | 2.16 | 0.00 | 0.01 | 136.34 | 29.83 | 2.21 | 0.00 | 0.00 |
| TraesCS3B01G390900 | 2624.10 | 880.86 | 1.57 | 0.00 | 0.00 | 3134.62 | 1237.25 | 1.34 | 0.00 | 0.00 |
| TraesCS3B01G396700 | 367.78 | 96.78 | 1.93 | 0.00 | 0.00 | 556.56 | 146.32 | 1.93 | 0.00 | 0.00 |
| TraesCS3B01G418800 | 7001.86 | 3093.77 | 1.18 | 0.00 | 0.03 | 7717.86 | 3235.90 | 1.25 | 0.00 | 0.00 |
| TraesCS3B01G524500 | 601.49 | 118.52 | 2.35 | 0.00 | 0.00 | 528.65 | 187.98 | 1.50 | 0.00 | 0.05 |
| TraesCS3D01G129800 | 1217.30 | 607.34 | 1.00 | 0.00 | 0.04 | 1524.44 | 725.66 | 1.07 | 0.00 | 0.00 |
| TraesCS3D01G350800 | 70.89 | 20.88 | 1.78 | 0.00 | 0.03 | 139.14 | 29.96 | 2.23 | 0.00 | 0.01 |
| TraesCS3D01G352100 | 1929.14 | 646.66 | 1.58 | 0.00 | 0.00 | 2629.88 | 1024.42 | 1.36 | 0.00 | 0.00 |
| TraesCS3D01G379700 | 1773.89 | 711.13 | 1.32 | 0.00 | 0.04 | 3134.52 | 1052.40 | 1.58 | 0.00 | 0.00 |
| TraesCS3D01G393900 | 929.77 | 219.85 | 2.08 | 0.00 | 0.00 | 1325.39 | 392.10 | 1.76 | 0.00 | 0.00 |
| TraesCS4A01G048700 | 369.14 | 160.79 | 1.20 | 0.00 | 0.02 | 479.64 | 161.72 | 1.57 | 0.00 | 0.00 |
| TraesCS4A01G229600 | 1141.30 | 469.08 | 1.28 | 0.00 | 0.00 | 1387.85 | 594.69 | 1.22 | 0.00 | 0.00 |
| TraesCS4A01G234100 | 2981.24 | 1033.65 | 1.53 | 0.00 | 0.00 | 1988.72 | 845.03 | 1.23 | 0.00 | 0.00 |
| TraesCS4B01G081200 | 283.54 | 110.53 | 1.36 | 0.00 | 0.03 | 246.32 | 59.94 | 2.04 | 0.00 | 0.00 |
| TraesCS4B01G262200 | 157.11 | 66.25 | 1.24 | 0.00 | 0.02 | 181.54 | 81.06 | 1.16 | 0.00 | 0.02 |
| TraesCS4D01G006200 | 226.42 | 81.22 | 1.48 | 0.00 | 0.04 | 267.41 | 105.67 | 1.34 | 0.00 | 0.00 |
| TraesCS4D01G066400 | 3602.60 | 1501.13 | 1.26 | 0.00 | 0.04 | 3868.09 | 1552.48 | 1.32 | 0.00 | 0.01 |
| TraesCS4D01G079900 | 773.53 | 246.27 | 1.65 | 0.00 | 0.00 | 562.28 | 245.25 | 1.20 | 0.00 | 0.00 |
| TraesCS4D01G211900 | 394.89 | 139.05 | 1.50 | 0.00 | 0.00 | 386.16 | 145.32 | 1.41 | 0.00 | 0.00 |
| TraesCS4D01G262100 | 923.59 | 326.01 | 1.50 | 0.00 | 0.00 | 1090.84 | 406.13 | 1.43 | 0.00 | 0.00 |
| TraesCS5A01G209800 | 711.84 | 286.05 | 1.32 | 0.00 | 0.03 | 1207.36 | 464.99 | 1.38 | 0.00 | 0.00 |
| TraesCS5A01G355200 | 1290.64 | 375.84 | 1.78 | 0.00 | 0.00 | 1271.94 | 572.39 | 1.15 | 0.00 | 0.01 |
| TraesCS5B01G039100 | 521.61 | 195.01 | 1.42 | 0.00 | 0.01 | 401.99 | 157.54 | 1.35 | 0.00 | 0.00 |
| TraesCS5B01G053400 | 117.95 | 38.04 | 1.63 | 0.00 | 0.00 | 143.72 | 43.89 | 1.70 | 0.00 | 0.00 |
| TraesCS5B01G076400 | 835.45 | 374.81 | 1.16 | 0.00 | 0.00 | 839.04 | 410.79 | 1.03 | 0.00 | 0.02 |
| TraesCS5B01G130100 | 397.93 | 130.62 | 1.61 | 0.00 | 0.00 | 556.33 | 120.97 | 2.20 | 0.00 | 0.00 |
| TraesCS5B01G207900 | 731.61 | 291.40 | 1.33 | 0.00 | 0.04 | 987.13 | 459.02 | 1.11 | 0.00 | 0.05 |
| TraesCS5B01G357400 | 1848.96 | 553.26 | 1.74 | 0.00 | 0.00 | 1858.30 | 676.53 | 1.46 | 0.00 | 0.00 |
| TraesCS5D01G082700 | 750.30 | 305.84 | 1.29 | 0.00 | 0.00 | 778.27 | 363.75 | 1.10 | 0.00 | 0.00 |
| TraesCS5D01G111700 | 325.63 | 75.03 | 2.12 | 0.00 | 0.00 | 676.91 | 253.89 | 1.42 | 0.00 | 0.01 |
| TraesCS5D01G216100 | 962.28 | 350.15 | 1.46 | 0.00 | 0.03 | 1146.24 | 536.66 | 1.10 | 0.00 | 0.03 |
| TraesCS5D01G299600 | 425.12 | 158.24 | 1.42 | 0.00 | 0.00 | 421.56 | 137.65 | 1.62 | 0.00 | 0.00 |
| TraesCS5D01G362500 | 725.13 | 243.64 | 1.57 | 0.00 | 0.00 | 699.79 | 340.00 | 1.04 | 0.00 | 0.02 |
| TraesCS5D01G403700 | 1095.19 | 235.46 | 2.22 | 0.00 | 0.00 | 1874.77 | 580.41 | 1.69 | 0.00 | 0.00 |
| TraesCS6A01G341900 | 380.77 | 147.57 | 1.36 | 0.00 | 0.04 | 521.93 | 198.65 | 1.39 | 0.00 | 0.00 |
| TraesCS6A01G355100 | 116.89 | 45.88 | 1.35 | 0.00 | 0.02 | 157.43 | 59.56 | 1.40 | 0.00 | 0.00 |
| TraesCS6B01G048800 | 63.87 | 6.84 | 3.19 | 0.00 | 0.00 | 39.40 | 10.41 | 1.90 | 0.00 | 0.02 |
| TraesCS6B01G178500 | 826.05 | 321.79 | 1.36 | 0.00 | 0.00 | 1114.00 | 516.57 | 1.11 | 0.00 | 0.01 |
| TraesCS6B01G264500 | 689.99 | 308.60 | 1.16 | 0.00 | 0.01 | 568.80 | 270.04 | 1.07 | 0.00 | 0.02 |
| TraesCS6B01G278900 | 170.22 | 38.11 | 2.15 | 0.00 | 0.00 | 117.26 | 39.14 | 1.58 | 0.00 | 0.00 |
| TraesCS6D01G140100 | 504.40 | 248.99 | 1.02 | 0.00 | 0.02 | 769.35 | 357.93 | 1.10 | 0.00 | 0.00 |
| TraesCS6D01G218600 | 504.14 | 212.74 | 1.25 | 0.00 | 0.01 | 457.49 | 226.01 | 1.02 | 0.00 | 0.03 |
| TraesCS7A01G027200 | 90.75 | 25.93 | 1.81 | 0.00 | 0.00 | 87.57 | 33.02 | 1.41 | 0.00 | 0.03 |
| TraesCS7A01G053100 | 218.15 | 94.45 | 1.20 | 0.00 | 0.01 | 189.70 | 81.72 | 1.22 | 0.00 | 0.02 |
| TraesCS7A01G138200 | 1209.49 | 495.10 | 1.29 | 0.00 | 0.00 | 1414.62 | 667.08 | 1.08 | 0.00 | 0.00 |
| TraesCS7A01G213800 | 468.49 | 211.60 | 1.15 | 0.00 | 0.03 | 418.48 | 145.50 | 1.52 | 0.00 | 0.01 |
| TraesCS7A01G246000 | 72.37 | 25.63 | 1.48 | 0.00 | 0.04 | 108.64 | 31.19 | 1.82 | 0.00 | 0.00 |
| TraesCS7A01G330000 | 299.73 | 93.24 | 1.68 | 0.00 | 0.01 | 423.13 | 173.72 | 1.28 | 0.00 | 0.00 |
| TraesCS7A01G506100 | 1454.26 | 511.92 | 1.51 | 0.00 | 0.02 | 1201.16 | 556.05 | 1.11 | 0.00 | 0.04 |
| TraesCS7A01G518300 | 1175.03 | 497.35 | 1.24 | 0.00 | 0.01 | 1448.56 | 378.85 | 1.94 | 0.00 | 0.00 |
| TraesCS7B01G450100 | 1151.16 | 410.07 | 1.49 | 0.00 | 0.00 | 1785.13 | 554.89 | 1.69 | 0.00 | 0.00 |
| TraesCS7B01G455300 | 798.42 | 317.32 | 1.33 | 0.00 | 0.00 | 868.36 | 398.85 | 1.12 | 0.00 | 0.02 |
| TraesCS7D01G236600 | 66.40 | 20.85 | 1.67 | 0.00 | 0.00 | 66.40 | 20.85 | 1.67 | 0.00 | 0.01 |
| TraesCS7D01G249700 | 27.06 | 3.09 | 3.11 | 0.00 | 0.02 | 24.91 | 3.15 | 2.94 | 0.00 | 0.03 |
| TraesCS7D01G508200 | 167.31 | 45.85 | 1.87 | 0.00 | 0.00 | 315.11 | 45.57 | 2.80 | 0.00 | 0.00 |
| TraesCS7D01G512700 | 651.68 | 280.42 | 1.22 | 0.00 | 0.02 | 258.33 | 92.55 | 1.48 | 0.00 | 0.00 |
| TraesCSU01G110500 | 591.78 | 274.53 | 1.11 | 0.00 | 0.01 | 643.74 | 292.23 | 1.14 | 0.00 | 0.00 |
| TraesCSU01G121000 | 83.73 | 27.24 | 1.61 | 0.00 | 0.02 | 58.18 | 20.62 | 1.50 | 0.00 | 0.03 |
| TraesCSU01G122500 | 108.65 | 43.40 | 1.32 | 0.00 | 0.02 | 74.73 | 26.13 | 1.53 | 0.00 | 0.01 |
| Novel.11172 | 18.88 | 92.28 | -2.25 | 0.00 | 0.00 | 9.33 | 79.48 | -3.09 | 0.00 | 0.00 |
| Novel.11605 | 22.54 | 86.23 | -1.97 | 0.00 | 0.00 | 14.44 | 66.43 | -2.23 | 0.00 | 0.03 |
| Novel.15643 | 220.86 | 777.40 | -1.82 | 0.00 | 0.00 | 149.03 | 563.55 | -1.92 | 0.00 | 0.00 |
| Novel.16636 | 36.42 | 127.34 | -1.84 | 0.00 | 0.00 | 29.47 | 129.07 | -2.14 | 0.00 | 0.00 |
| Novel.17408 | 4.04 | 41.90 | -3.49 | 0.00 | 0.00 | 4.12 | 44.48 | -3.42 | 0.00 | 0.00 |
| Novel.21694 | 13.06 | 78.54 | -2.59 | 0.00 | 0.00 | 14.09 | 60.21 | -2.11 | 0.00 | 0.00 |
| Novel.31059 | 6.25 | 34.54 | -2.45 | 0.00 | 0.04 | 2.38 | 36.59 | -3.99 | 0.00 | 0.00 |
| Novel.31549 | 58.77 | 210.31 | -1.86 | 0.00 | 0.00 | 28.69 | 152.18 | -2.42 | 0.00 | 0.00 |
| Novel.34591 | 9.46 | 52.28 | -2.57 | 0.00 | 0.02 | 6.22 | 44.51 | -2.88 | 0.00 | 0.01 |
| Novel.38998 | 130.11 | 491.68 | -1.92 | 0.00 | 0.00 | 187.47 | 604.17 | -1.69 | 0.00 | 0.00 |
| Novel.40524 | 18.77 | 64.79 | -1.76 | 0.00 | 0.01 | 7.19 | 50.84 | -2.84 | 0.00 | 0.00 |
| Novel.4956 | 1772.38 | 5714.89 | -1.69 | 0.00 | 0.00 | 2101.97 | 5495.70 | -1.39 | 0.00 | 0.04 |
| Novel.49888 | 223.69 | 1039.63 | -2.22 | 0.00 | 0.00 | 203.95 | 1060.59 | -2.38 | 0.00 | 0.00 |
| Novel.51281 | 57.11 | 224.31 | -2.00 | 0.00 | 0.00 | 43.85 | 122.72 | -1.50 | 0.00 | 0.03 |
| Novel.52343 | 57.18 | 221.08 | -1.96 | 0.00 | 0.00 | 51.69 | 164.86 | -1.68 | 0.00 | 0.00 |
| Novel.52910 | 169.53 | 522.26 | -1.63 | 0.00 | 0.01 | 312.16 | 910.72 | -1.55 | 0.00 | 0.00 |
| Novel.52911 | 34.31 | 162.43 | -2.28 | 0.00 | 0.00 | 25.72 | 187.48 | -2.88 | 0.00 | 0.00 |
| Novel.53136 | 43.44 | 144.01 | -1.75 | 0.00 | 0.00 | 20.05 | 102.29 | -2.37 | 0.00 | 0.00 |
| Novel.55143 | 5.09 | 76.16 | -4.01 | 0.00 | 0.00 | 4.67 | 52.11 | -3.45 | 0.00 | 0.00 |
| Novel.55540 | 33.42 | 139.19 | -2.08 | 0.00 | 0.01 | 46.96 | 123.02 | -1.39 | 0.00 | 0.00 |
| Novel.55541 | 51.27 | 201.70 | -1.98 | 0.00 | 0.00 | 108.61 | 319.17 | -1.56 | 0.00 | 0.00 |
| Novel.56624 | 4.99 | 44.53 | -3.18 | 0.00 | 0.00 | 3.12 | 34.09 | -3.52 | 0.00 | 0.00 |
| Novel.58170 | 7.18 | 36.86 | -2.27 | 0.00 | 0.03 | 7.68 | 48.83 | -2.70 | 0.00 | 0.00 |
| Novel.8271 | 14.68 | 62.76 | -2.10 | 0.00 | 0.03 | 8.22 | 43.12 | -2.42 | 0.00 | 0.02 |
| TraesCS1A01G015400 | 91.63 | 359.75 | -1.99 | 0.00 | 0.00 | 50.46 | 218.85 | -2.13 | 0.00 | 0.00 |
| TraesCS1A01G043300 | 12.95 | 97.52 | -2.98 | 0.00 | 0.00 | 5.99 | 73.70 | -3.64 | 0.00 | 0.00 |
| TraesCS1A01G048700 | 50.02 | 214.16 | -2.11 | 0.00 | 0.00 | 63.37 | 151.60 | -1.26 | 0.00 | 0.00 |
| TraesCS1A01G065500 | 90.90 | 210.28 | -1.22 | 0.00 | 0.04 | 69.44 | 206.18 | -1.58 | 0.00 | 0.00 |
| TraesCS1A01G069300 | 412.37 | 1090.40 | -1.41 | 0.00 | 0.01 | 281.48 | 680.46 | -1.27 | 0.00 | 0.00 |
| TraesCS1A01G129400 | 48.63 | 116.21 | -1.27 | 0.00 | 0.03 | 61.42 | 160.48 | -1.39 | 0.00 | 0.00 |
| TraesCS1A01G141900 | 70.45 | 202.87 | -1.54 | 0.00 | 0.00 | 54.89 | 128.03 | -1.22 | 0.00 | 0.00 |
| TraesCS1A01G203200 | 305.22 | 2113.98 | -2.79 | 0.00 | 0.00 | 139.70 | 1352.61 | -3.28 | 0.00 | 0.00 |
| TraesCS1A01G217000 | 48.48 | 140.78 | -1.55 | 0.00 | 0.01 | 59.10 | 128.28 | -1.12 | 0.00 | 0.02 |
| TraesCS1A01G223600 | 430.56 | 1128.30 | -1.39 | 0.00 | 0.04 | 463.23 | 1003.01 | -1.12 | 0.00 | 0.01 |
| TraesCS1A01G252400 | 34.47 | 144.82 | -2.07 | 0.00 | 0.00 | 41.31 | 122.51 | -1.56 | 0.00 | 0.00 |
| TraesCS1A01G261400 | 101.48 | 242.62 | -1.27 | 0.00 | 0.02 | 119.34 | 262.40 | -1.14 | 0.00 | 0.00 |
| TraesCS1A01G342900 | 37.79 | 125.46 | -1.75 | 0.00 | 0.01 | 26.29 | 80.42 | -1.61 | 0.00 | 0.01 |
| TraesCS1A01G370100 | 202.54 | 535.83 | -1.41 | 0.00 | 0.04 | 109.65 | 268.14 | -1.30 | 0.00 | 0.00 |
| TraesCS1A01G400700 | 17.61 | 79.77 | -2.19 | 0.00 | 0.00 | 11.49 | 64.78 | -2.53 | 0.00 | 0.01 |
| TraesCS1B01G201500 | 16.36 | 97.18 | -2.63 | 0.00 | 0.03 | 0.66 | 41.48 | -6.01 | 0.00 | 0.00 |
| TraesCS1B01G217200 | 273.40 | 1270.28 | -2.22 | 0.00 | 0.00 | 231.16 | 656.63 | -1.51 | 0.00 | 0.00 |
| TraesCS1B01G272200 | 28.70 | 92.60 | -1.71 | 0.00 | 0.00 | 46.16 | 98.18 | -1.09 | 0.00 | 0.05 |
| TraesCS1B01G272900 | 185.07 | 577.92 | -1.64 | 0.00 | 0.00 | 202.56 | 515.65 | -1.35 | 0.00 | 0.00 |
| TraesCS1B01G276700 | 24.13 | 112.05 | -2.18 | 0.00 | 0.02 | 53.61 | 183.52 | -1.78 | 0.00 | 0.00 |
| TraesCS1B01G360700 | 14.93 | 109.44 | -2.87 | 0.00 | 0.00 | 7.79 | 74.17 | -3.23 | 0.00 | 0.00 |
| TraesCS1B01G433700 | 6.57 | 55.98 | -3.22 | 0.00 | 0.01 | 3.06 | 48.62 | -4.04 | 0.00 | 0.00 |
| TraesCS1D01G013300 | 33.33 | 137.68 | -2.04 | 0.00 | 0.00 | 17.73 | 94.63 | -2.43 | 0.00 | 0.00 |
| TraesCS1D01G075500 | 672.70 | 1650.28 | -1.30 | 0.00 | 0.00 | 504.98 | 1126.27 | -1.16 | 0.00 | 0.00 |
| TraesCS1D01G101900 | 287.66 | 1059.42 | -1.88 | 0.00 | 0.00 | 45.99 | 369.34 | -3.01 | 0.00 | 0.00 |
| TraesCS1D01G141000 | 109.45 | 258.92 | -1.25 | 0.00 | 0.02 | 102.99 | 240.27 | -1.22 | 0.00 | 0.00 |
| TraesCS1D01G153900 | 830.20 | 2691.62 | -1.70 | 0.00 | 0.00 | 898.75 | 2049.53 | -1.19 | 0.00 | 0.00 |
| TraesCS1D01G164300 | 49.14 | 242.56 | -2.31 | 0.00 | 0.00 | 91.17 | 275.02 | -1.59 | 0.00 | 0.01 |
| TraesCS1D01G177400 | 143.41 | 548.41 | -1.95 | 0.00 | 0.00 | 66.99 | 351.23 | -2.40 | 0.00 | 0.00 |
| TraesCS1D01G189400 | 122.38 | 440.72 | -1.86 | 0.00 | 0.02 | 49.43 | 279.93 | -2.51 | 0.00 | 0.00 |
| TraesCS1D01G218800 | 11.23 | 69.01 | -2.62 | 0.00 | 0.00 | 30.49 | 78.53 | -1.37 | 0.00 | 0.01 |
| TraesCS2A01G103300 | 10.76 | 44.28 | -2.02 | 0.00 | 0.01 | 12.91 | 50.65 | -1.97 | 0.00 | 0.01 |
| TraesCS2A01G104500 | 111.58 | 419.10 | -1.92 | 0.00 | 0.00 | 124.99 | 334.43 | -1.42 | 0.00 | 0.00 |
| TraesCS2A01G132600 | 348.20 | 1373.89 | -1.98 | 0.00 | 0.00 | 605.72 | 1316.45 | -1.12 | 0.00 | 0.01 |
| TraesCS2A01G138000 | 17.36 | 81.09 | -2.26 | 0.00 | 0.00 | 21.59 | 89.74 | -2.06 | 0.00 | 0.00 |
| TraesCS2A01G189600 | 233.61 | 722.30 | -1.62 | 0.00 | 0.00 | 177.38 | 416.43 | -1.23 | 0.00 | 0.00 |
| TraesCS2A01G192500 | 8.92 | 40.36 | -2.12 | 0.00 | 0.02 | 12.31 | 42.52 | -1.78 | 0.00 | 0.03 |
| TraesCS2A01G211200 | 8.55 | 67.14 | -3.07 | 0.00 | 0.00 | 2.95 | 29.07 | -3.32 | 0.00 | 0.00 |
| TraesCS2A01G266900 | 240.62 | 901.83 | -1.90 | 0.00 | 0.00 | 585.35 | 1443.01 | -1.30 | 0.00 | 0.00 |
| TraesCS2A01G267900 | 58.78 | 288.20 | -2.29 | 0.00 | 0.00 | 64.76 | 216.44 | -1.74 | 0.00 | 0.00 |
| TraesCS2A01G352000 | 175.12 | 838.35 | -2.27 | 0.00 | 0.00 | 115.60 | 559.87 | -2.28 | 0.00 | 0.00 |
| TraesCS2A01G398500 | 478.85 | 1170.64 | -1.29 | 0.00 | 0.00 | 587.01 | 1435.14 | -1.29 | 0.00 | 0.00 |
| TraesCS2A01G522100 | 76.36 | 293.99 | -1.93 | 0.00 | 0.00 | 351.11 | 790.15 | -1.17 | 0.00 | 0.00 |
| TraesCS2A01G525000 | 30.32 | 128.77 | -2.10 | 0.00 | 0.00 | 48.57 | 121.02 | -1.33 | 0.00 | 0.02 |
| TraesCS2A01G530900 | 1483.70 | 5287.11 | -1.83 | 0.00 | 0.00 | 1100.42 | 3432.85 | -1.64 | 0.00 | 0.00 |
| TraesCS2B01G110300 | 108.93 | 343.23 | -1.67 | 0.00 | 0.01 | 60.35 | 275.17 | -2.19 | 0.00 | 0.00 |
| TraesCS2B01G131200 | 151.25 | 942.95 | -2.65 | 0.00 | 0.00 | 51.31 | 555.40 | -3.44 | 0.00 | 0.00 |
| TraesCS2B01G131300 | 94.80 | 437.92 | -2.22 | 0.00 | 0.00 | 68.48 | 253.45 | -1.88 | 0.00 | 0.00 |
| TraesCS2B01G182500 | 8.34 | 38.81 | -2.26 | 0.00 | 0.01 | 14.38 | 81.83 | -2.52 | 0.00 | 0.00 |
| TraesCS2B01G182700 | 63.76 | 162.88 | -1.37 | 0.00 | 0.04 | 56.92 | 122.48 | -1.10 | 0.00 | 0.01 |
| TraesCS2B01G217500 | 161.54 | 361.71 | -1.16 | 0.00 | 0.02 | 43.43 | 135.35 | -1.64 | 0.00 | 0.00 |
| TraesCS2B01G268300 | 94.00 | 346.94 | -1.89 | 0.00 | 0.00 | 78.62 | 328.32 | -2.06 | 0.00 | 0.00 |
| TraesCS2B01G275500 | 83.72 | 332.81 | -1.98 | 0.00 | 0.00 | 211.85 | 542.74 | -1.36 | 0.00 | 0.00 |
| TraesCS2B01G359400 | 368.62 | 1166.16 | -1.67 | 0.00 | 0.00 | 117.63 | 600.76 | -2.36 | 0.00 | 0.00 |
| TraesCS2B01G387500 | 28.53 | 147.72 | -2.36 | 0.00 | 0.00 | 22.40 | 100.07 | -2.18 | 0.00 | 0.00 |
| TraesCS2B01G389600 | 103.07 | 229.79 | -1.16 | 0.00 | 0.02 | 107.13 | 222.25 | -1.05 | 0.00 | 0.02 |
| TraesCS2B01G491000 | 12.03 | 50.18 | -2.12 | 0.00 | 0.00 | 13.26 | 48.00 | -1.87 | 0.00 | 0.03 |
| TraesCS2B01G560100 | 560.56 | 2761.23 | -2.30 | 0.00 | 0.00 | 397.75 | 1585.33 | -2.00 | 0.00 | 0.00 |
| TraesCS2D01G093500 | 427.67 | 1336.87 | -1.65 | 0.00 | 0.00 | 291.81 | 1109.52 | -1.93 | 0.00 | 0.00 |
| TraesCS2D01G110000 | 49.91 | 221.08 | -2.16 | 0.00 | 0.00 | 18.59 | 103.27 | -2.49 | 0.00 | 0.00 |
| TraesCS2D01G141000 | 22.79 | 80.15 | -1.84 | 0.00 | 0.00 | 24.10 | 66.60 | -1.45 | 0.00 | 0.05 |
| TraesCS2D01G168800 | 30.69 | 89.39 | -1.52 | 0.00 | 0.02 | 8.68 | 95.25 | -3.48 | 0.00 | 0.00 |
| TraesCS2D01G217400 | 34.90 | 150.67 | -2.15 | 0.00 | 0.00 | 31.47 | 96.10 | -1.61 | 0.00 | 0.00 |
| TraesCS2D01G256200 | 44.94 | 216.23 | -2.30 | 0.00 | 0.00 | 34.39 | 199.21 | -2.54 | 0.00 | 0.00 |
| TraesCS2D01G257100 | 24.34 | 176.43 | -2.83 | 0.00 | 0.00 | 100.39 | 307.99 | -1.62 | 0.00 | 0.00 |
| TraesCS2D01G323500 | 13.09 | 54.45 | -2.01 | 0.00 | 0.01 | 26.37 | 66.03 | -1.33 | 0.00 | 0.05 |
| TraesCS2D01G339900 | 2.31 | 137.77 | -5.88 | 0.00 | 0.04 | 2.43 | 83.58 | -5.13 | 0.00 | 0.03 |
| TraesCS2D01G370400 | 8.48 | 53.64 | -2.60 | 0.00 | 0.05 | 9.16 | 50.22 | -2.45 | 0.00 | 0.02 |
| TraesCS2D01G396000 | 710.09 | 1636.47 | -1.21 | 0.00 | 0.00 | 1009.48 | 2230.90 | -1.14 | 0.00 | 0.01 |
| TraesCS2D01G401300 | 43.92 | 140.12 | -1.66 | 0.00 | 0.00 | 45.47 | 115.46 | -1.35 | 0.00 | 0.03 |
| TraesCS2D01G432300 | 9.25 | 56.70 | -2.62 | 0.00 | 0.00 | 6.79 | 45.04 | -2.77 | 0.00 | 0.00 |
| TraesCS2D01G453800 | 24.18 | 73.90 | -1.61 | 0.00 | 0.01 | 29.61 | 78.49 | -1.41 | 0.00 | 0.04 |
| TraesCS2D01G476900 | 2.31 | 25.90 | -3.66 | 0.00 | 0.00 | 1.49 | 24.12 | -4.13 | 0.00 | 0.03 |
| TraesCS2D01G487100 | 14.14 | 54.53 | -1.95 | 0.00 | 0.01 | 31.99 | 81.31 | -1.36 | 0.00 | 0.04 |
| TraesCS2D01G524200 | 22.92 | 95.74 | -2.07 | 0.00 | 0.00 | 60.72 | 198.65 | -1.72 | 0.00 | 0.01 |
| TraesCS3A01G025600 | 15.27 | 96.61 | -2.72 | 0.00 | 0.00 | 3.55 | 61.72 | -4.21 | 0.00 | 0.00 |
| TraesCS3A01G140700 | 139.47 | 401.92 | -1.53 | 0.00 | 0.00 | 161.17 | 446.79 | -1.47 | 0.00 | 0.00 |
| TraesCS3A01G180700 | 307.35 | 623.31 | -1.02 | 0.00 | 0.04 | 244.58 | 527.48 | -1.11 | 0.00 | 0.00 |
| TraesCS3A01G207900 | 65.49 | 210.08 | -1.70 | 0.00 | 0.00 | 38.57 | 102.41 | -1.42 | 0.00 | 0.05 |
| TraesCS3A01G217000 | 42.24 | 138.04 | -1.70 | 0.00 | 0.00 | 61.26 | 168.55 | -1.45 | 0.00 | 0.02 |
| TraesCS3A01G277200 | 29.20 | 147.74 | -2.33 | 0.00 | 0.00 | 16.64 | 108.51 | -2.72 | 0.00 | 0.00 |
| TraesCS3A01G277300 | 78.47 | 200.78 | -1.36 | 0.00 | 0.01 | 58.91 | 191.69 | -1.71 | 0.00 | 0.00 |
| TraesCS3A01G290300 | 26.78 | 120.11 | -2.18 | 0.00 | 0.00 | 41.61 | 155.15 | -1.91 | 0.00 | 0.00 |
| TraesCS3A01G325100 | 67.21 | 173.73 | -1.38 | 0.00 | 0.01 | 23.14 | 115.14 | -2.34 | 0.00 | 0.00 |
| TraesCS3A01G336800 | 796.01 | 2406.39 | -1.60 | 0.00 | 0.00 | 92.87 | 367.32 | -1.99 | 0.00 | 0.00 |
| TraesCS3A01G372100 | 118.24 | 338.47 | -1.51 | 0.00 | 0.00 | 179.11 | 360.39 | -1.01 | 0.00 | 0.03 |
| TraesCS3A01G394500 | 50.72 | 163.66 | -1.69 | 0.00 | 0.00 | 62.55 | 139.43 | -1.15 | 0.00 | 0.02 |
| TraesCS3A01G426200 | 24.99 | 70.69 | -1.51 | 0.00 | 0.02 | 14.32 | 61.22 | -2.10 | 0.00 | 0.00 |
| TraesCS3A01G489100 | 334.68 | 1041.81 | -1.64 | 0.00 | 206.38 | 578.22 | -1.49 | 0.00 | 0.01 |  |
| TraesCS3A01G517400 | 120.02 | 316.13 | -1.41 | 0.00 | 0.03 | 72.25 | 194.74 | -1.44 | 0.00 | 0.00 |
| TraesCS3B01G112000 | 44.18 | 127.60 | -1.54 | 0.00 | 0.02 | 49.02 | 113.89 | -1.22 | 0.00 | 0.05 |
| TraesCS3B01G210400 | 39.11 | 198.30 | -2.36 | 0.00 | 0.00 | 44.43 | 179.59 | -2.02 | 0.00 | 0.00 |
| TraesCS3B01G211400 | 77.24 | 227.07 | -1.57 | 0.00 | 0.00 | 20.32 | 94.58 | -2.21 | 0.00 | 0.00 |
| TraesCS3B01G272700 | 16.59 | 147.12 | -3.21 | 0.00 | 0.00 | 6.82 | 48.49 | -2.89 | 0.00 | 0.01 |
| TraesCS3B01G293000 | 245.03 | 570.73 | -1.22 | 0.00 | 0.01 | 268.76 | 612.67 | -1.19 | 0.00 | 0.00 |
| TraesCS3B01G325100 | 113.57 | 278.32 | -1.30 | 0.00 | 0.04 | 128.93 | 335.06 | -1.38 | 0.00 | 0.00 |
| TraesCS3B01G367900 | 320.89 | 854.12 | -1.42 | 0.00 | 0.01 | 259.54 | 628.01 | -1.28 | 0.00 | 0.01 |
| TraesCS3B01G438500 | 105.19 | 248.61 | -1.25 | 0.00 | 0.01 | 98.14 | 251.27 | -1.36 | 0.00 | 0.00 |
| TraesCS3B01G438800 | 64.26 | 193.83 | -1.61 | 0.00 | 0.02 | 29.55 | 82.65 | -1.49 | 0.00 | 0.05 |
| TraesCS3D01G020100 | 17.82 | 235.45 | -3.70 | 0.00 | 0.00 | 2.98 | 170.64 | -5.87 | 0.00 | 0.04 |
| TraesCS3D01G185600 | 72.02 | 174.70 | -1.27 | 0.00 | 0.03 | 60.69 | 158.60 | -1.38 | 0.00 | 0.00 |
| TraesCS3D01G210800 | 116.38 | 268.67 | -1.22 | 0.00 | 0.04 | 73.93 | 174.99 | -1.25 | 0.00 | 0.01 |
| TraesCS3D01G215500 | 15.73 | 80.33 | -2.39 | 0.00 | 0.00 | 14.84 | 71.35 | -2.29 | 0.00 | 0.01 |
| TraesCS3D01G225700 | 12.50 | 57.87 | -2.19 | 0.00 | 0.00 | 19.90 | 65.18 | -1.71 | 0.00 | 0.01 |
| TraesCS3D01G244900 | 6.92 | 56.92 | -3.12 | 0.00 | 0.05 | 5.81 | 54.91 | -3.27 | 0.00 | 0.00 |
| TraesCS3D01G282600 | 852.47 | 2437.02 | -1.52 | 0.00 | 0.00 | 719.44 | 1548.34 | -1.11 | 0.00 | 0.00 |
| TraesCS3D01G416700 | 21.80 | 300.23 | -3.76 | 0.00 | 0.00 | 52.69 | 551.46 | -3.39 | 0.00 | 0.00 |
| TraesCS3D01G484000 | 197.44 | 502.36 | -1.35 | 0.00 | 0.01 | 183.02 | 382.43 | -1.07 | 0.00 | 0.03 |
| TraesCS4A01G005700 | 27.73 | 100.89 | -1.82 | 0.00 | 0.01 | 30.67 | 86.69 | -1.50 | 0.00 | 0.01 |
| TraesCS4A01G017200 | 134.85 | 503.39 | -1.91 | 0.00 | 0.00 | 36.89 | 306.27 | -3.07 | 0.00 | 0.00 |
| TraesCS4A01G017400 | 9.18 | 57.82 | -2.63 | 0.00 | 0.04 | 27.14 | 91.86 | -1.75 | 0.00 | 0.02 |
| TraesCS4A01G050800 | 326.88 | 912.47 | -1.49 | 0.00 | 0.02 | 111.11 | 523.70 | -2.24 | 0.00 | 0.00 |
| TraesCS4A01G102900 | 77.02 | 364.66 | -2.24 | 0.00 | 0.00 | 148.86 | 402.95 | -1.44 | 0.00 | 0.00 |
| TraesCS4A01G118200 | 60.20 | 189.68 | -1.65 | 0.00 | 0.00 | 37.25 | 214.83 | -2.53 | 0.00 | 0.00 |
| TraesCS4A01G129800 | 16.42 | 59.69 | -1.90 | 0.00 | 0.04 | 6.47 | 52.89 | -3.04 | 0.00 | 0.00 |
| TraesCS4A01G167400 | 10.43 | 46.18 | -2.15 | 0.00 | 0.02 | 8.42 | 40.89 | -2.29 | 0.00 | 0.00 |
| TraesCS4A01G179400 | 87.20 | 404.32 | -2.22 | 0.00 | 0.00 | 106.48 | 313.72 | -1.56 | 0.00 | 0.00 |
| TraesCS4A01G180200 | 187.84 | 641.36 | -1.77 | 0.00 | 0.00 | 145.08 | 385.32 | -1.41 | 0.00 | 0.00 |
| TraesCS4A01G250400 | 13.82 | 68.07 | -2.26 | 0.00 | 0.04 | 49.60 | 287.63 | -2.54 | 0.00 | 0.00 |
| TraesCS4A01G293000 | 184.27 | 524.03 | -1.50 | 0.00 | 0.02 | 316.67 | 852.48 | -1.43 | 0.00 | 0.00 |
| TraesCS4A01G404800 | 182.72 | 951.13 | -2.38 | 0.00 | 0.00 | 117.57 | 785.48 | -2.74 | 0.00 | 0.00 |
| TraesCS4B01G031500 | 844.81 | 2046.29 | -1.28 | 0.00 | 0.00 | 963.83 | 1955.72 | -1.02 | 0.00 | 0.00 |
| TraesCS4B01G186200 | 13.89 | 60.19 | -2.07 | 0.00 | 0.02 | 3.21 | 49.77 | -4.05 | 0.00 | 0.00 |
| TraesCS4B01G200900 | 167.61 | 352.82 | -1.08 | 0.00 | 0.05 | 188.06 | 379.92 | -1.02 | 0.00 | 0.00 |
| TraesCS4B01G201600 | 71.60 | 384.44 | -2.44 | 0.00 | 0.00 | 92.14 | 354.63 | -1.95 | 0.00 | 0.00 |
| TraesCS4B01G233100 | 207.89 | 454.49 | -1.13 | 0.00 | 0.04 | 202.62 | 414.44 | -1.03 | 0.00 | 0.00 |
| TraesCS4B01G304600 | 77.98 | 234.95 | -1.58 | 0.00 | 0.02 | 56.69 | 274.30 | -2.28 | 0.00 | 0.00 |
| TraesCS4B01G356600 | 289.37 | 1196.15 | -2.05 | 0.00 | 0.00 | 168.50 | 1355.52 | -3.01 | 0.00 | 0.00 |
| TraesCS4D01G095800 | 99.40 | 228.33 | -1.21 | 0.00 | 0.01 | 82.93 | 241.52 | -1.55 | 0.00 | 0.00 |
| TraesCS4D01G103600 | 10.86 | 57.79 | -2.47 | 0.00 | 0.00 | 10.68 | 51.86 | -2.30 | 0.00 | 0.00 |
| TraesCS4D01G134300 | 90.46 | 213.93 | -1.23 | 0.00 | 0.02 | 124.32 | 262.31 | -1.08 | 0.00 | 0.00 |
| TraesCS4D01G187300 | 31.34 | 115.74 | -1.88 | 0.00 | 0.01 | 9.48 | 107.42 | -3.52 | 0.00 | 0.00 |
| TraesCS4D01G246300 | 37.43 | 137.77 | -1.91 | 0.00 | 0.01 | 33.61 | 93.76 | -1.47 | 0.00 | 0.01 |
| TraesCS4D01G273600 | 161.88 | 369.15 | -1.20 | 0.00 | 0.03 | 116.56 | 243.05 | -1.06 | 0.00 | 0.00 |
| TraesCS4D01G285600 | 177.77 | 380.37 | -1.09 | 0.00 | 0.05 | 97.56 | 357.87 | -1.88 | 0.00 | 0.00 |
| TraesCS4D01G302800 | 157.70 | 686.18 | -2.12 | 0.00 | 0.00 | 115.14 | 728.54 | -2.67 | 0.00 | 0.00 |
| TraesCS4D01G322000 | 227.52 | 487.05 | -1.10 | 0.00 | 0.01 | 189.33 | 418.96 | -1.15 | 0.00 | 0.00 |
| TraesCS4D01G350300 | 194.56 | 685.31 | -1.82 | 0.00 | 0.00 | 103.43 | 583.80 | -2.50 | 0.00 | 0.00 |
| TraesCS5A01G093600 | 6.73 | 54.23 | -3.04 | 0.00 | 0.00 | 20.58 | 100.71 | -2.29 | 0.00 | 0.00 |
| TraesCS5A01G145500 | 10.28 | 54.41 | -2.47 | 0.00 | 0.00 | 5.36 | 33.48 | -2.67 | 0.00 | 0.01 |
| TraesCS5A01G148100 | 49.88 | 128.49 | -1.39 | 0.00 | 0.03 | 33.34 | 102.41 | -1.63 | 0.00 | 0.01 |
| TraesCS5A01G163900 | 4.50 | 43.10 | -3.39 | 0.00 | 0.00 | 3.38 | 34.03 | -3.37 | 0.00 | 0.00 |
| TraesCS5A01G212000 | 166.53 | 449.85 | -1.44 | 0.00 | 0.00 | 110.89 | 234.81 | -1.08 | 0.00 | 0.01 |
| TraesCS5A01G262400 | 10.53 | 64.63 | -2.66 | 0.00 | 0.01 | 7.53 | 71.40 | -3.26 | 0.00 | 0.00 |
| TraesCS5A01G263500 | 302.31 | 658.59 | -1.12 | 0.00 | 0.01 | 180.69 | 552.64 | -1.61 | 0.00 | 0.00 |
| TraesCS5A01G394900 | 105.74 | 423.37 | -1.99 | 0.00 | 0.00 | 245.73 | 491.33 | -1.00 | 0.00 | 0.01 |
| TraesCS5A01G401600 | 32.45 | 157.77 | -2.25 | 0.00 | 0.00 | 110.41 | 287.62 | -1.38 | 0.00 | 0.00 |
| TraesCS5A01G407000 | 490.93 | 1216.78 | -1.31 | 0.00 | 0.02 | 366.17 | 1402.35 | -1.94 | 0.00 | 0.00 |
| TraesCS5A01G526000 | 138.88 | 541.09 | -1.96 | 0.00 | 0.00 | 51.67 | 533.96 | -3.38 | 0.00 | 0.00 |
| TraesCS5B01G022400 | 34.79 | 204.69 | -2.59 | 0.00 | 0.00 | 30.56 | 184.04 | -2.60 | 0.00 | 0.00 |
| TraesCS5B01G063300 | 165.41 | 379.63 | -1.20 | 0.00 | 0.00 | 174.20 | 376.47 | -1.11 | 0.00 | 0.03 |
| TraesCS5B01G068200 | 86.78 | 273.46 | -1.66 | 0.00 | 0.00 | 45.14 | 171.77 | -1.94 | 0.00 | 0.01 |
| TraesCS5B01G099700 | 223.80 | 666.29 | -1.58 | 0.00 | 0.02 | 161.72 | 429.58 | -1.41 | 0.00 | 0.00 |
| TraesCS5B01G219700 | 165.10 | 518.28 | -1.65 | 0.00 | 0.00 | 292.74 | 732.68 | -1.33 | 0.00 | 0.03 |
| TraesCS5B01G252100 | 1.74 | 28.78 | -3.95 | 0.00 | 0.00 | 4.47 | 33.98 | -2.98 | 0.00 | 0.00 |
| TraesCS5B01G260800 | 39.72 | 144.35 | -1.87 | 0.00 | 0.00 | 46.01 | 124.15 | -1.43 | 0.00 | 0.02 |
| TraesCS5B01G262900 | 223.83 | 472.61 | -1.07 | 0.00 | 0.05 | 202.98 | 412.15 | -1.02 | 0.00 | 0.05 |
| TraesCS5B01G266200 | 101.80 | 265.52 | -1.38 | 0.00 | 0.00 | 127.15 | 255.63 | -1.01 | 0.00 | 0.04 |
| TraesCS5B01G373300 | 48.34 | 182.02 | -1.94 | 0.00 | 0.01 | 16.95 | 77.50 | -2.21 | 0.00 | 0.00 |
| TraesCS5B01G383500 | 42.86 | 188.27 | -2.17 | 0.00 | 0.00 | 31.16 | 112.55 | -1.87 | 0.00 | 0.00 |
| TraesCS5B01G392900 | 15.84 | 75.86 | -2.32 | 0.00 | 0.00 | 16.98 | 60.51 | -1.83 | 0.00 | 0.01 |
| TraesCS5B01G405500 | 15.74 | 96.45 | -2.60 | 0.00 | 0.00 | 20.59 | 85.76 | -2.06 | 0.00 | 0.00 |
| TraesCS5B01G406300 | 40.37 | 171.54 | -2.07 | 0.00 | 0.00 | 161.76 | 324.67 | -1.00 | 0.00 | 0.01 |
| TraesCS5B01G440500 | 10.03 | 65.10 | -2.78 | 0.00 | 0.01 | 4.84 | 56.45 | -3.55 | 0.00 | 0.00 |
| TraesCS5B01G478100 | 74.66 | 451.80 | -2.59 | 0.00 | 0.00 | 185.11 | 779.98 | -2.08 | 0.00 | 0.00 |
| TraesCS5D01G105900 | 22.77 | 115.53 | -2.39 | 0.00 | 0.00 | 6.96 | 56.17 | -3.04 | 0.00 | 0.00 |
| TraesCS5D01G133700 | 5.55 | 28.53 | -2.42 | 0.00 | 0.02 | 28.55 | 160.86 | -2.50 | 0.00 | 0.00 |
| TraesCS5D01G199500 | 301.40 | 1582.00 | -2.40 | 0.00 | 0.00 | 67.71 | 611.98 | -3.19 | 0.00 | 0.00 |
| TraesCS5D01G240600 | 29.12 | 99.92 | -1.80 | 0.00 | 0.02 | 18.70 | 111.66 | -2.59 | 0.00 | 0.00 |
| TraesCS5D01G253500 | 19.43 | 147.01 | -2.94 | 0.00 | 0.01 | 19.61 | 67.19 | -1.78 | 0.00 | 0.03 |
| TraesCS5D01G270000 | 3.12 | 54.30 | -4.19 | 0.00 | 0.00 | 1.80 | 51.18 | -4.75 | 0.00 | 0.00 |
| TraesCS5D01G270300 | 30.62 | 118.42 | -1.97 | 0.00 | 0.03 | 2.18 | 71.59 | -5.18 | 0.00 | 0.00 |
| TraesCS5D01G321800 | 13.69 | 66.80 | -2.29 | 0.00 | 0.02 | 10.60 | 46.25 | -2.15 | 0.00 | 0.05 |
| TraesCS5D01G322900 | 37.69 | 124.16 | -1.70 | 0.00 | 0.01 | 64.42 | 295.52 | -2.20 | 0.00 | 0.00 |
| TraesCS5D01G380600 | 53.02 | 312.97 | -2.58 | 0.00 | 0.00 | 18.47 | 137.22 | -2.91 | 0.00 | 0.00 |
| TraesCS5D01G389400 | 248.57 | 643.67 | -1.38 | 0.00 | 0.00 | 209.28 | 511.08 | -1.29 | 0.00 | 0.00 |
| TraesCS5D01G404300 | 96.31 | 363.24 | -1.91 | 0.00 | 0.00 | 120.65 | 365.57 | -1.60 | 0.00 | 0.00 |
| TraesCS5D01G438900 | 28.99 | 146.35 | -2.38 | 0.00 | 0.00 | 14.03 | 77.95 | -2.49 | 0.00 | 0.00 |
| TraesCS6A01G002400 | 2.44 | 27.91 | -3.46 | 0.00 | 0.01 | 2.75 | 20.96 | -2.98 | 0.00 | 0.04 |
| TraesCS6A01G046800 | 68.15 | 196.34 | -1.54 | 0.00 | 0.05 | 19.73 | 80.35 | -2.05 | 0.00 | 0.00 |
| TraesCS6A01G115400 | 314.78 | 670.99 | -1.09 | 0.00 | 0.00 | 229.36 | 629.16 | -1.46 | 0.00 | 0.03 |
| TraesCS6A01G157000 | 13.64 | 58.22 | -2.09 | 0.00 | 0.01 | 5.96 | 33.10 | -2.50 | 0.00 | 0.00 |
| TraesCS6A01G170200 | 7.75 | 58.03 | -2.91 | 0.00 | 0.00 | 2.92 | 51.36 | -4.15 | 0.00 | 0.00 |
| TraesCS6A01G222300 | 14.30 | 81.05 | -2.57 | 0.00 | 0.01 | 7.79 | 38.44 | -2.32 | 0.00 | 0.01 |
| TraesCS6A01G266100 | 221.65 | 642.35 | -1.53 | 0.00 | 0.00 | 91.06 | 591.48 | -2.71 | 0.00 | 0.00 |
| TraesCS6A01G284700 | 48.26 | 154.19 | -1.68 | 0.00 | 0.00 | 43.20 | 133.46 | -1.63 | 0.00 | 0.00 |
| TraesCS6B01G007400 | 3.12 | 22.73 | -2.93 | 0.00 | 0.03 | 1.92 | 25.93 | -3.75 | 0.00 | 0.00 |
| TraesCS6B01G062900 | 134.42 | 446.70 | -1.74 | 0.00 | 0.00 | 83.01 | 327.17 | -1.98 | 0.00 | 0.00 |
| TraesCS6B01G198400 | 31.22 | 166.41 | -2.43 | 0.00 | 0.00 | 23.33 | 99.26 | -2.09 | 0.00 | 0.00 |
| TraesCS6B01G199900 | 101.80 | 509.76 | -2.33 | 0.00 | 0.00 | 81.31 | 332.45 | -2.04 | 0.00 | 0.00 |
| TraesCS6B01G226700 | 123.26 | 299.65 | -1.29 | 0.00 | 0.01 | 113.69 | 292.61 | -1.36 | 0.00 | 0.00 |
| TraesCS6B01G258400 | 44.62 | 216.80 | -2.30 | 0.00 | 0.01 | 29.90 | 103.89 | -1.81 | 0.00 | 0.00 |
| TraesCS6B01G276100 | 43.73 | 122.37 | -1.51 | 0.00 | 0.01 | 46.18 | 102.67 | -1.15 | 0.00 | 0.04 |
| TraesCS6B01G313400 | 42.81 | 125.50 | -1.55 | 0.00 | 0.04 | 50.36 | 140.45 | -1.48 | 0.00 | 0.00 |
| TraesCS6B01G387700 | 206.50 | 772.10 | -1.91 | 0.00 | 0.00 | 493.44 | 1095.13 | -1.15 | 0.00 | 0.00 |
| TraesCS6B01G420000 | 345.79 | 1187.93 | -1.78 | 0.00 | 0.02 | 123.30 | 611.02 | -2.31 | 0.00 | 0.00 |
| TraesCS6B01G439700 | 13.65 | 64.19 | -2.27 | 0.00 | 0.00 | 6.70 | 47.72 | -2.87 | 0.00 | 0.00 |
| TraesCS6D01G053800 | 101.24 | 353.81 | -1.82 | 0.00 | 0.00 | 60.76 | 364.04 | -2.59 | 0.00 | 0.00 |
| TraesCS6D01G054000 | 107.49 | 308.98 | -1.53 | 0.00 | 0.01 | 62.16 | 264.55 | -2.10 | 0.00 | 0.00 |
| TraesCS6D01G157800 | 26.93 | 93.56 | -1.84 | 0.00 | 0.00 | 11.05 | 80.80 | -2.90 | 0.00 | 0.00 |
| TraesCS6D01G160000 | 17.58 | 84.66 | -2.27 | 0.00 | 0.00 | 8.25 | 81.86 | -3.30 | 0.00 | 0.00 |
| TraesCS6D01G161400 | 91.15 | 533.64 | -2.56 | 0.00 | 0.00 | 78.99 | 368.65 | -2.23 | 0.00 | 0.00 |
| TraesCS6D01G216600 | 27.05 | 124.85 | -2.25 | 0.00 | 0.00 | 15.20 | 82.22 | -2.44 | 0.00 | 0.00 |
| TraesCS6D01G288000 | 361.61 | 981.18 | -1.44 | 0.00 | 0.00 | 328.31 | 741.81 | -1.18 | 0.00 | 0.03 |
| TraesCS6D01G293300 | 157.56 | 428.26 | -1.45 | 0.00 | 0.00 | 162.07 | 459.13 | -1.50 | 0.00 | 0.00 |
| TraesCS7A01G033100 | 338.74 | 959.04 | -1.51 | 0.00 | 0.01 | 293.25 | 738.18 | -1.33 | 0.00 | 0.00 |
| TraesCS7A01G147300 | 291.50 | 818.64 | -1.49 | 0.00 | 0.00 | 220.49 | 760.09 | -1.79 | 0.00 | 0.00 |
| TraesCS7A01G215900 | 52.68 | 139.54 | -1.39 | 0.00 | 0.03 | 41.85 | 102.79 | -1.29 | 0.00 | 0.05 |
| TraesCS7A01G228600 | 11.45 | 67.04 | -2.55 | 0.00 | 0.00 | 14.09 | 47.89 | -1.78 | 0.00 | 0.02 |
| TraesCS7A01G249400 | 12.37 | 64.88 | -2.44 | 0.00 | 0.00 | 7.76 | 41.99 | -2.45 | 0.00 | 0.00 |
| TraesCS7A01G392300 | 6.83 | 54.51 | -2.91 | 0.00 | 0.00 | 11.57 | 77.45 | -2.75 | 0.00 | 0.00 |
| TraesCS7A01G394600 | 156.00 | 685.15 | -2.14 | 0.00 | 0.00 | 134.94 | 622.34 | -2.21 | 0.00 | 0.00 |
| TraesCS7A01G394700 | 205.45 | 567.05 | -1.47 | 0.00 | 0.00 | 145.62 | 478.63 | -1.72 | 0.00 | 0.00 |
| TraesCS7A01G398900 | 5.44 | 26.23 | -2.27 | 0.00 | 0.03 | 1.69 | 48.32 | -4.89 | 0.00 | 0.00 |
| TraesCS7A01G450400 | 215.50 | 519.77 | -1.27 | 0.00 | 0.04 | 74.23 | 292.50 | -1.99 | 0.00 | 0.00 |
| TraesCS7A01G544500 | 8251.65 | 17048.71 | -1.05 | 0.00 | 0.05 | 5784.99 | 13255.88 | -1.20 | 0.00 | 0.01 |
| TraesCS7B01G084600 | 109.28 | 589.22 | -2.44 | 0.00 | 0.00 | 124.53 | 390.97 | -1.65 | 0.00 | 0.00 |
| TraesCS7B01G194900 | 20.27 | 85.70 | -2.08 | 0.00 | 0.00 | 27.06 | 92.76 | -1.78 | 0.00 | 0.00 |
| TraesCS7B01G227900 | 10.86 | 62.56 | -2.59 | 0.00 | 0.00 | 12.49 | 57.89 | -2.24 | 0.00 | 0.00 |
| TraesCS7B01G247900 | 28.76 | 208.99 | -2.89 | 0.00 | 0.00 | 9.94 | 58.66 | -2.58 | 0.00 | 0.01 |
| TraesCS7B01G285600 | 93.02 | 429.96 | -2.22 | 0.00 | 0.00 | 65.03 | 254.75 | -1.98 | 0.00 | 0.00 |
| TraesCS7B01G294200 | 55.95 | 180.58 | -1.67 | 0.00 | 0.00 | 110.87 | 308.15 | -1.47 | 0.00 | 0.00 |
| TraesCS7B01G328400 | 617.86 | 1922.58 | -1.64 | 0.00 | 0.02 | 244.08 | 1133.75 | -2.22 | 0.00 | 0.00 |
| TraesCS7B01G350500 | 115.14 | 350.18 | -1.60 | 0.00 | 0.00 | 209.32 | 521.42 | -1.32 | 0.00 | 0.00 |
| TraesCS7B01G382000 | 12.31 | 71.67 | -2.49 | 0.00 | 0.01 | 34.73 | 122.35 | -1.82 | 0.00 | 0.00 |
| TraesCS7B01G433800 | 312.13 | 937.42 | -1.59 | 0.00 | 0.01 | 270.14 | 687.87 | -1.35 | 0.00 | 0.00 |
| TraesCS7D01G009400 | 30.43 | 280.75 | -3.23 | 0.00 | 0.00 | 17.47 | 118.63 | -2.79 | 0.00 | 0.00 |
| TraesCS7D01G125500 | 31.49 | 110.91 | -1.81 | 0.00 | 0.00 | 45.79 | 111.79 | -1.29 | 0.00 | 0.01 |
| TraesCS7D01G137800 | 34.18 | 152.45 | -2.19 | 0.00 | 0.00 | 7.50 | 91.86 | -3.66 | 0.00 | 0.00 |
| TraesCS7D01G163100 | 548.86 | 1685.35 | -1.62 | 0.00 | 0.00 | 503.07 | 1422.94 | -1.50 | 0.00 | 0.00 |
| TraesCS7D01G163200 | 22.36 | 70.90 | -1.66 | 0.00 | 0.01 | 13.17 | 59.35 | -2.16 | 0.00 | 0.01 |
| TraesCS7D01G170600 | 32.37 | 126.88 | -2.00 | 0.00 | 0.02 | 21.34 | 79.52 | -1.92 | 0.00 | 0.00 |
| TraesCS7D01G181600 | 173.84 | 509.72 | -1.55 | 0.00 | 0.03 | 113.10 | 315.32 | -1.48 | 0.00 | 0.00 |
| TraesCS7D01G209800 | 3.00 | 50.26 | -4.32 | 0.00 | 0.00 | 1.89 | 43.15 | -4.50 | 0.00 | 0.00 |
| TraesCS7D01G229700 | 8.43 | 96.74 | -3.56 | 0.00 | 0.00 | 7.56 | 52.74 | -2.84 | 0.00 | 0.00 |
| TraesCS7D01G233400 | 26.44 | 108.09 | -2.08 | 0.00 | 0.01 | 25.57 | 90.35 | -1.83 | 0.00 | 0.00 |
| TraesCS7D01G263600 | 25.09 | 87.88 | -1.83 | 0.00 | 0.00 | 29.89 | 74.44 | -1.32 | 0.00 | 0.02 |
| TraesCS7D01G267300 | 25.79 | 111.83 | -2.15 | 0.00 | 0.00 | 15.43 | 81.26 | -2.38 | 0.00 | 0.00 |
| TraesCS7D01G284400 | 76.78 | 167.48 | -1.12 | 0.00 | 0.02 | 99.29 | 202.64 | -1.03 | 0.00 | 0.01 |
| TraesCS7D01G312200 | 17.71 | 90.41 | -2.40 | 0.00 | 0.00 | 16.66 | 57.03 | -1.78 | 0.00 | 0.01 |
| TraesCS7D01G365500 | 4.88 | 36.94 | -2.82 | 0.00 | 0.02 | 9.80 | 45.45 | -2.24 | 0.00 | 0.01 |
| TraesCS7D01G379300 | 51.46 | 165.72 | -1.69 | 0.00 | 0.00 | 39.14 | 113.51 | -1.54 | 0.00 | 0.00 |
| TraesCS7D01G387600 | 75.45 | 225.59 | -1.57 | 0.00 | 0.01 | 115.02 | 296.63 | -1.37 | 0.00 | 0.00 |
| TraesCS7D01G387700 | 16.31 | 186.49 | -3.52 | 0.00 | 0.00 | 20.22 | 234.41 | -3.55 | 0.00 | 0.00 |
| TraesCS7D01G387800 | 14.59 | 107.25 | -2.87 | 0.00 | 0.00 | 32.56 | 134.38 | -2.06 | 0.00 | 0.00 |
| TraesCS7D01G390100 | 80.68 | 209.36 | -1.39 | 0.00 | 0.03 | 60.83 | 162.35 | -1.43 | 0.00 | 0.00 |
| TraesCS7D01G420500 | 593.10 | 1644.03 | -1.47 | 0.00 | 0.03 | 382.30 | 1388.50 | -1.86 | 0.00 | 0.00 |
| TraesCS7D01G439800 | 100.86 | 302.47 | -1.58 | 0.00 | 0.02 | 38.26 | 183.57 | -2.27 | 0.00 | 0.00 |
| TraesCS7D01G463700 | 43.69 | 166.06 | -1.96 | 0.00 | 0.00 | 19.10 | 87.59 | -2.19 | 0.00 | 0.00 |
| TraesCS7D01G466800 | 23.21 | 110.62 | -2.21 | 0.00 | 0.01 | 90.09 | 231.96 | -1.37 | 0.00 | 0.03 |
| TraesCSU01G074200 | 11.21 | 89.91 | -3.09 | 0.00 | 0.00 | 9.02 | 53.21 | -2.56 | 0.00 | 0.00 |
