# Supplementary figures and images for: Transcriptomic and Metabolomics Analysis of Different Endosperm Region under Nitrogen Treatments
Source: Int J Mol Sci. 2019 Aug 28;20(17):4212. doi: 10.3390/ijms20174212 (PMC6747615; doi:10.3390/ijms20174212)

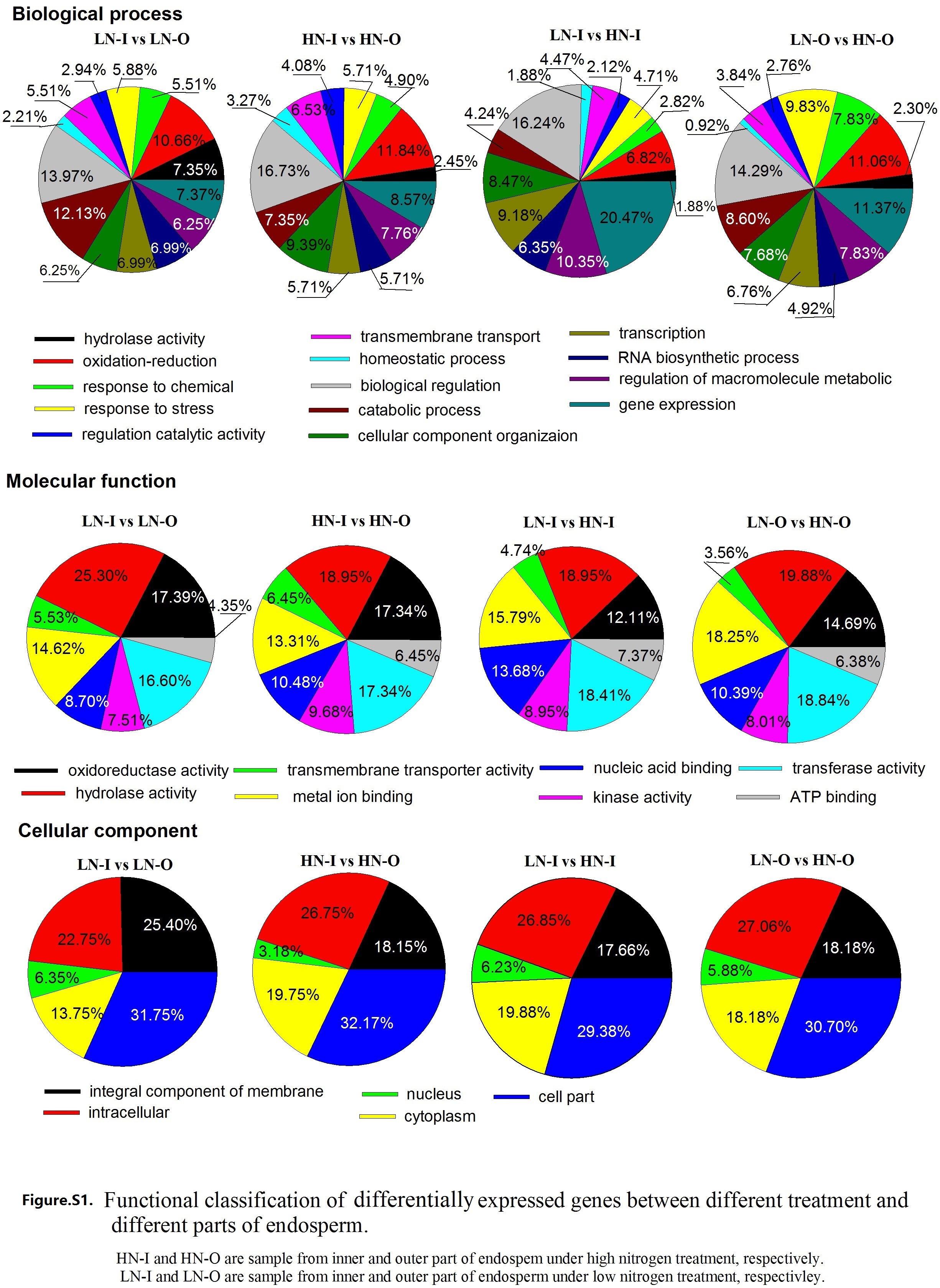

Supplement: Supplementary file 1 [file ijms-20-04212-s001.zip › ijms-575327-for final-suppl/Supplementary-Figure S1.JPG]

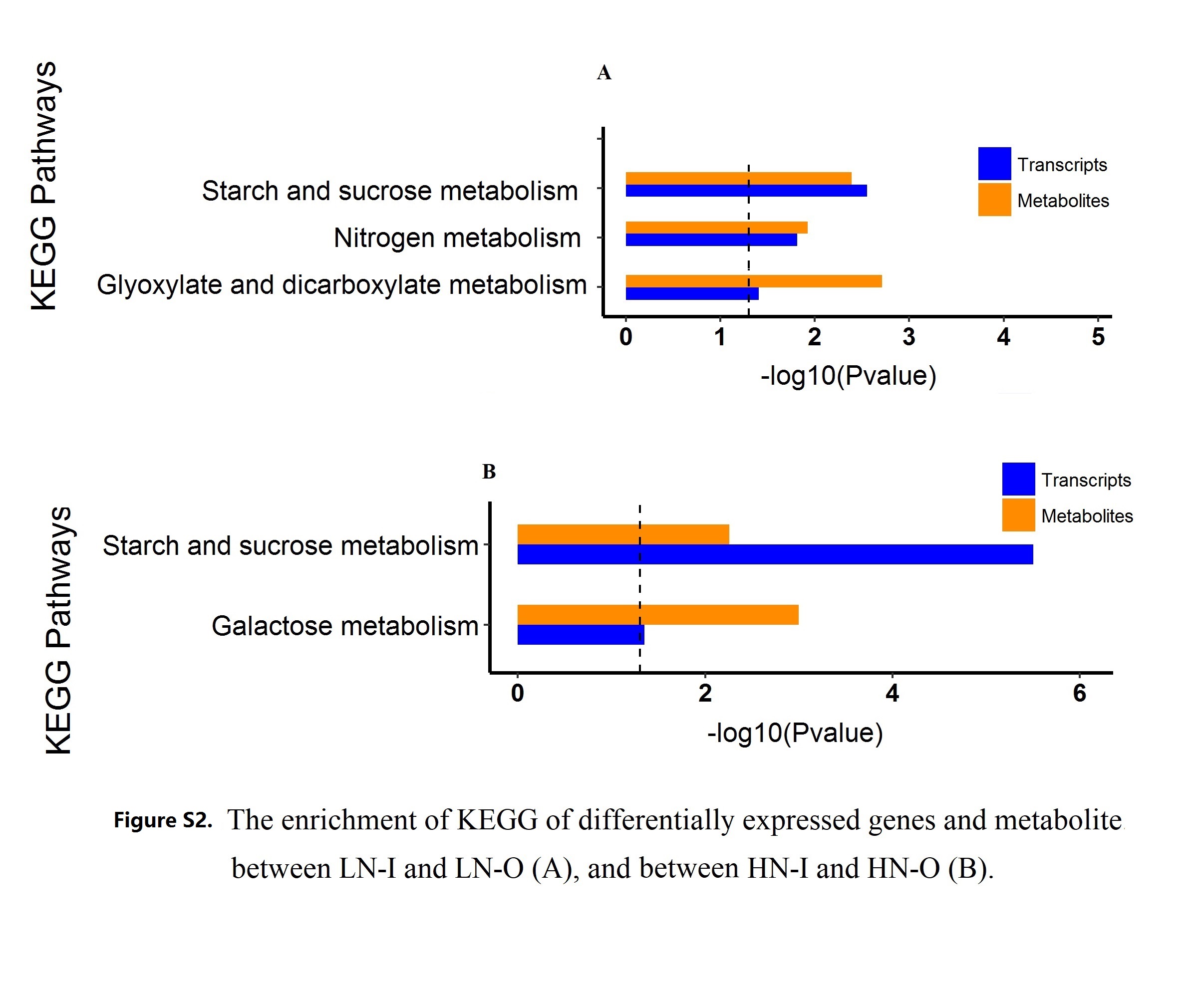

Supplement: Supplementary file 1 [file ijms-20-04212-s001.zip › ijms-575327-for final-suppl/Supplementary-Figure S2.jpg]

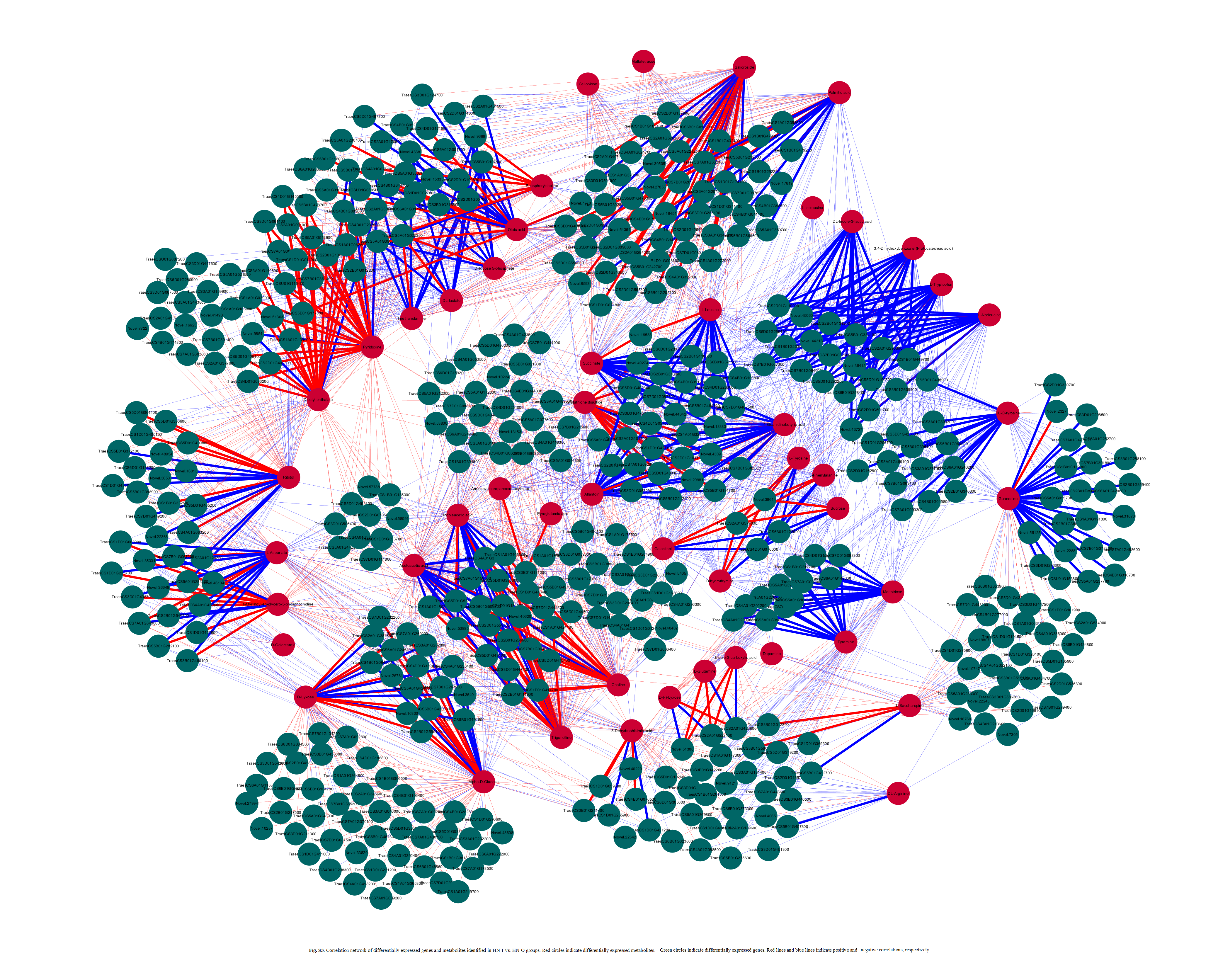

Supplement: Supplementary file 1 [file ijms-20-04212-s001.zip › ijms-575327-for final-suppl/Supplementary-Figure S3.png]

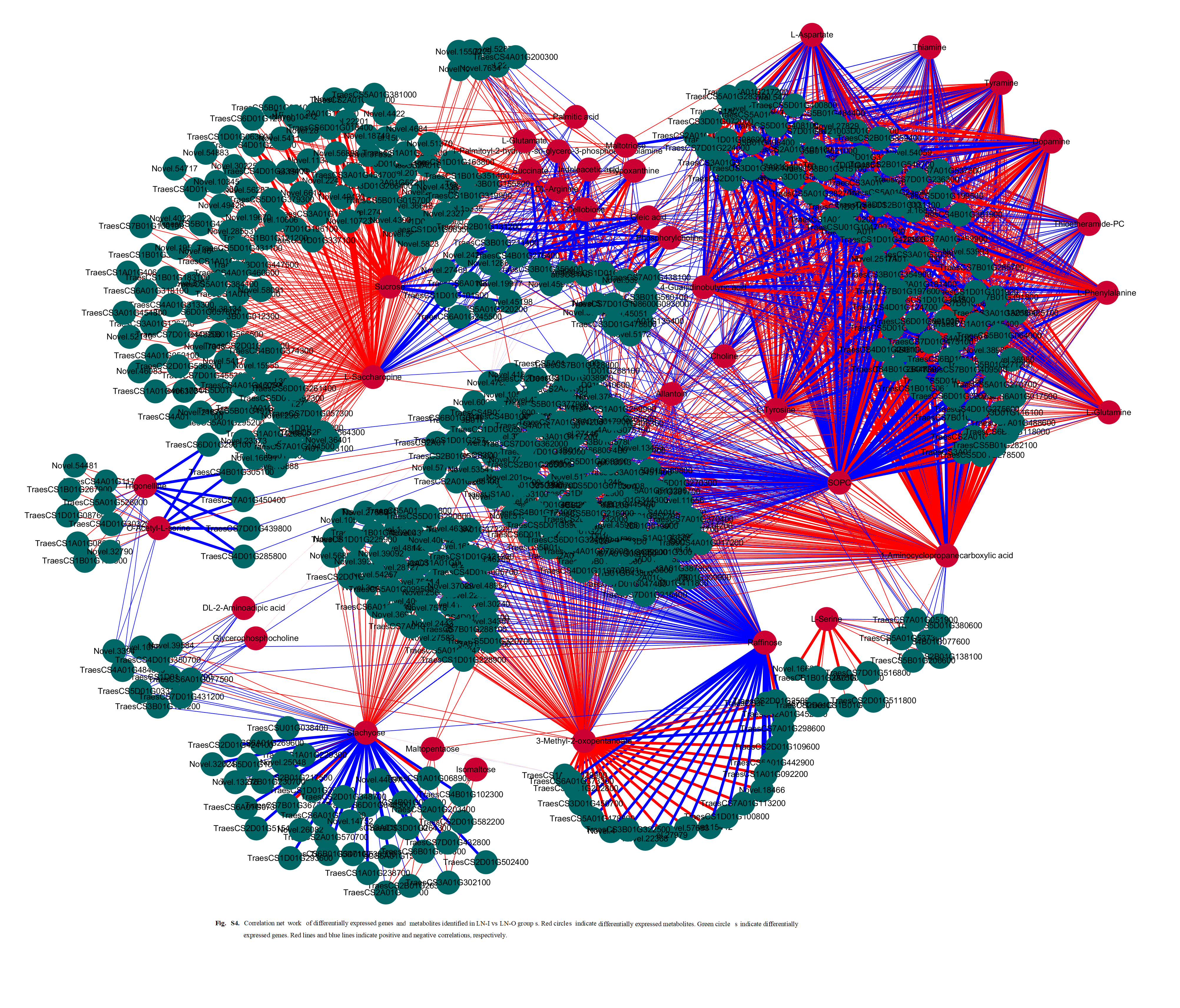

Supplement: Supplementary file 1 [file ijms-20-04212-s001.zip › ijms-575327-for final-suppl/Supplementary-Figure S4.png]
