# Supplementary material for: Transcriptomic and Metabolomics Analysis of Different Endosperm Region under Nitrogen Treatments
Source: Int J Mol Sci. 2019 Aug 28;20(17):4212. doi: 10.3390/ijms20174212 (PMC6747615; doi:10.3390/ijms20174212)
Supplement: Supplementary file 1 [file ijms-20-04212-s001.zip › ijms-575327-for final-suppl/Supplementary-Table S2.doc]

**Table S2-I** Differently expressed genes between LN-I and LN-O related to carbon and nitrogen metabolism

| **Gene_ID** | **LN-I**  **Readcount** | **LN-O**  **Readcount** | **log2FoldChange** | ***p*-Value** | **Padj** | **GO Annotation** |  |
| --- | --- | --- | --- | --- | --- | --- | --- |
| TraesCS6A01G017500 | 176.07 | 39.89 | 2.14 | 5E-09 | 3E-05 | nitrate reductase (NADH) activity |  |
| TraesCS6D01G020700 | 14.27 | 1.18 | 3.61 | 3E-04 | 5E-02 | Nitrate reductase |  |
| TraesCS4B01G077600 | 498.19 | 208.04 | 1.26 | 1E-04 | 3E-02 | serine-type endopeptidase activity |  |
| TraesCS4D01G076000 | 329.55 | 104.04 | 1.66 | 1E-06 | 2E-03 | serine-type endopeptidase activity |  |
| TraesCS5A01G526000 | 151.43 | 47.33 | 1.69 | 2E-04 | 4E-02 | serine-type endopeptidase activity |  |
| TraesCS1D01G395200 | 30.06 | 2.78 | 3.39 | 7E-06 | 5E-03 | serine-type endopeptidase activity |  |
| TraesCS3B01G515100 | 12139.8 | 3184.88 | 1.93 | 1E-06 | 2E-03 | serine-type endopeptidase activity |  |
| TraesCS2A01G338800 | 354.69 | 160.03 | 1.14 | 3E-04 | 5E-02 | amino acid transmembrane transport |  |
| TraesCS1A01G191800 | 163.23 | 47.78 | 1.77 | 7E-05 | 2E-02 | amino acid transmembrane transport |  |
| TraesCS1A01G153100 | 512.7 | 237.52 | 1.11 | 2E-04 | 4E-02 | glutathione S-transferase activity |  |
| TraesCS3A01G302100 | 282.32 | 83.88 | 1.75 | 2E-04 | 4E-02 | Glutathione S-transferase |  |
| TraesCS1B01G043800 | 931.18 | 434.52 | 1.10 | 3E-04 | 5E-02 | Proteinase inhibitor |  |
| TraesCS1D01G059500 | 74.17 | 9.34 | 2.99 | 1E-06 | 2E-03 | EGF-type aspartate/asparagine hydroxylation |  |
| TraesCS6A01G048900 | 74.89 | 17.11 | 2.12 | 4E-05 | 1E-02 | Alpha-gliadin |  |
| TraesCS2A01G382700 | 130.1 | 33.09 | 1.97 | 2E-04 | 3E-02 | threonine aldolase |  |
| TraesCS1A01G425100 | 275.94 | 649.48 | -1.23 | 6E-05 | 2E-02 | Indole-3-acetic acid-amido synthetase |  |
| TraesCS1A01G086100 | 283.1 | 1900.78 | -2.75 | 3E-09 | 2E-05 | serine-type carboxypeptidase |  |
| TraesCS1D01G087600 | 130.82 | 830.82 | -2.67 | 2E-10 | 4E-06 | serine-type carboxypeptidase |  |
| TraesCS1B01G104500 | 52.45 | 217.01 | -2.06 | 5E-07 | 9E-04 | serine-type carboxypeptidase |  |
| TraesCS5D01G196500 | 8.97 | 38.3 | -2.07 | 1E-04 | 3E-02 | serine carboxypeptidase |  |
| TraesCS2D01G109600 | 34.46 | 163.47 | -2.22 | 2E-04 | 4E-02 | serine-type endopeptidase activity |  |
| TraesCS3B01G322500 | 2.28 | 27.63 | -3.52 | 2E-05 | 1E-02 | serine-type endopeptidase activity |  |
| TraesCS2D01G109400 | 205.01 | 767.57 | -1.90 | 2E-04 | 4E-02 | aspartic-type peptidase activity |  |
| TraesCS2A01G545700 | 118.47 | 350.99 | -1.56 | 2E-05 | 1E-02 | cysteine-type peptidase activity |  |
| TraesCS2D01G546600 | 91.69 | 253.24 | -1.45 | 3E-04 | 5E-02 | Cysteine proteinase |  |
| TraesCS1A01G406300 | 43.97 | 148.65 | -1.74 | 3E-06 | 3E-03 | iron-sulfur transferase activity |  |
| TraesCS3D01G034200 | 409.51 | 1471.73 | -1.84 | 2E-04 | 4E-02 | Trypsin inhibitor |  |
| TraesCS2B01G551300 | 8.21 | 40.7 | -2.28 | 2E-04 | 4E-02 | transmembrane amino acid transporter activity |  |
| TraesCS2A01G348600 | 937.86 | 3220.76 | -1.78 | 2E-04 | 4E-02 | Transmembrane amino acid transporter protein |  |
| TraesCS7A01G194500 | 35.85 | 275.8 | -2.92 | 1E-07 | 3E-04 | Transmembrane amino acid transporter protein |  |
| TraesCS7B01G100100 | 20.43 | 106.88 | -2.33 | 1E-05 | 6E-03 | Transmembrane amino acid transporter protein |  |
| TraesCS7D01G196100 | 20.44 | 148.04 | -2.81 | 2E-06 | 2E-03 | Transmembrane amino acid transporter protein |  |
| TraesCS1B01G064600 | 24.27 | 102.35 | -2.06 | 0.00 | 0.02 | Glycosyl hydrolases family |  |
| TraesCS4B01G344300 | 226.58 | 680.44 | -1.58 | 0.00 | 0.03 | Beta-amylase |  |
| TraesCS4D01G339400 | 258.18 | 1223.96 | -2.24 | 0.00 | 0.00 | Beta-amylase |  |
| TraesCS5A01G513200 | 370.53 | 1256.33 | -1.76 | 0.00 | 0.01 | Beta-amylase |  |
| TraesCS4B01G344700 | 1230.13 | 3461.1 | -1.49 | 0.00 | 0.01 | polygalacturonase activity |  |
| TraesCS6B01G118000 | 7.86 | 64.89 | -2.97 | 0.00 | 0.00 | polygalacturonase activity |  |
| TraesCS7A01G370400 | 53.46 | 119.51 | -1.16 | 0.00 | 0.04 | UDP-glucosyltransferase activity |  |
| TraesCS7B01G285700 | 40.92 | 146.83 | -1.82 | 0.00 | 0.03 | Glucan endo-1,3-beta-glucosidase |  |
| TraesCS2A01G161200 | 83.08 | 14.36 | 2.53 | 0.00 | 0.00 | Trehalose 6-phosphate phosphatase |  |
| TraesCS2D01G351300 | 122.66 | 39.81 | 1.62 | 0.00 | 0.04 | Endoglucanase |  |
| TraesCS3B01G211400 | 83.91 | 18.68 | 2.14 | 0.00 | 0.00 | Geranyl reductase |  |
| TraesCS3D01G264300 | 132.89 | 40.86 | 1.70 | 0.00 | 0.03 | UDP-glucosyltransferase activity |  |
| TraesCS5A01G248900 | 74.89 | 17.11 | 2.12 | 0.00 | 0.01 | malate transmembrane transport |  |
| TraesCS5B01G064000 | 79.15 | 19.46 | 2.01 | 0.00 | 0.01 | Glucan 1,3-beta-glucosidase |  |
| TraesCS7A01G298600 | 975.32 | 286.17 | 1.77 | 0.00 | 0.00 | cellulose synthase (UDP-forming) activity |  |
| TraesCS7D01G364900 | 748.35 | 291.25 | 1.36 | 0.00 | 0.03 | Myo-inositol oxygenase |  |
| TraesCS7D01G419900 | 52.6 | 2.87 | 4.20 | 0.00 | 0.00 | Xyloglucan endotransglucosylase |  |
| TraesCSU01G005100 | 101.73 | 36.38 | 1.48 | 0.00 | 0.04 | Glycosyltransferase |  |

**Table S2-II** Differently expressed genes between HN-I and HN-O related to carbon and nitrogen metabolism

| **Gene-ID** | **HN-I**  **Readcount** | **HN-O**  **Readcount** | **log2FoldChange** | ***p-*Value** | **Padj** |  |
| --- | --- | --- | --- | --- | --- | --- |
| TraesCS1A01G188900 | 197.93 | 72.56 | 1.45 | 0.00 | 0.00 | serine-type endopeptidase activity |
| TraesCS4D01G076000 | 243.56 | 94.07 | 1.37 | 0.00 | 0.01 | serine-type endopeptidase activity |
| TraesCS7A01G477900 | 201.17 | 21.44 | 3.23 | 0.00 | 0.00 | Glutathione S-transferase |
| TraesCS5A01G221200 | 2138.47 | 835.88 | 1.36 | 0.00 | 0.00 | S-adenosylmethionine decarboxylase |
| TraesCS1A01G370100 | 585.28 | 245.73 | 1.25 | 0.00 | 0.00 | glutathione dehydrogenase activity |
| TraesCS1D01G027800 | 2631.83 | 1290.99 | 1.03 | 0.00 | 0.01 | peptidase activity |
| TraesCS5D01G425800 | 16089.67 | 7242.37 | 1.15 | 0.00 | 0.03 | serine protease inhibitor |
| TraesCS5B01G478300 | 158.2 | 46.48 | 1.77 | 0.00 | 0.01 | serine protease inhibitor |
| TraesCS5A01G359700 | 4964.36 | 2037.28 | 1.29 | 0.00 | 0.02 | serine protease inhibitor |
| TraesCS6B01G099300 | 188.91 | 91.15 | 1.05 | 0.00 | 0.04 | ATP-dependent peptidase activity |
| TraesCS3B01G515100 | 12357.5 | 2894.58 | 2.09 | 0.00 | 0.00 | Aspartic peptidase |
| TraesCS5D01G556600 | 445.81 | 207.36 | 1.11 | 0.00 | 0.02 | N,N-dimethylaniline monooxygenase activityN， |
| TraesCSU01G153800 | 1088.54 | 491.96 | 1.15 | 0.00 | 0.04 | Alpha-gliadin |
| TraesCS2D01G135700 | 57.28 | 5.27 | 3.41 | 0.00 | 0.00 | serine-type carboxypeptidase |
| TraesCS4D01G184900 | 205.43 | 76.35 | 1.43 | 0.00 | 0.03 | Nicotianamine synthase |
| TraesCS6A01G245100 | 625.41 | 227.97 | 1.46 | 0.00 | 0.01 | transporter activity |
| TraesCS5A01G256600 | 102.73 | 22.93 | 2.16 | 0.00 | 0.00 | amino acid import |
| TraesCS5A01G521500 | 164.65 | 53.55 | 1.62 | 0.00 | 0.00 | sulfur compound transmembrane transporter activity |
| TraesCS7D01G351300 | 92.84 | 340.61 | -1.87 | 0.00 | 0.01 | 2S globulin |
| TraesCS1D01G265900 | 648.4 | 1628.53 | -1.33 | 0.00 | 0.00 | serine-type endopeptidase inhibitor activity |
| TraesCS3A01G046000 | 213.15 | 556.61 | -1.38 | 0.00 | 0.00 | serine protease inhibitor |
| TraesCS2A01G124200 | 30.15 | 85.52 | -1.51 | 0.00 | 0.02 | negative regulation of nitrogen compound metabolic process |
| TraesCS2D01G109600 | 25.7 | 96.6 | -1.91 | 0.00 | 0.04 | aspartic-type peptidase activity |
| TraesCS3D01G467300 | 25946.38 | 52683.3 | -1.02 | 0.00 | 0.04 | Aspartic peptidase |
| TraesCS7D01G060100 | 9.09 | 44.66 | -2.30 | 0.00 | 0.02 | cysteine-type peptidase activity |
| TraesCS1A01G406300 | 330.41 | 978.47 | -1.57 | 0.00 | 0.00 | iron-sulfur transferase activity |
| TraesCS1D01G414700 | 209.02 | 658.26 | -1.65 | 0.00 | 0.02 | metallo-sulfur cluster assembly |
| TraesCS4D01G171800 | 5489.93 | 11272.55 | -1.04 | 0.00 | 0.02 | nutrient reservoir activity cupin（globulins） |
| TraesCS1D01G296600 | 1.02 | 23.99 | -4.52 | 0.00 | 0.02 | ammonium transmembrane transport |
| TraesCS1B01G449700 | 18234.84 | 7177.27 | 1.35 | 0.00 | 0.00 | glucose-1-phosphate adenylyltransferase activity |
| TraesCS1B01G119300 | 209.58 | 100.2 | 1.07 | 0.00 | 0.02 | starch binding |
| TraesCS1D01G427400 | 24542.01 | 10942.71 | 1.17 | 0.00 | 0.01 | glucose-1-phosphate adenylyltransferase activity |
| TraesCS2A01G343600 | 714.59 | 223.78 | 1.68 | 0.00 | 0.00 | aldose 1-epimerase activity |
| TraesCS3A01G159900 | 277.44 | 135.9 | 1.03 | 0.00 | 0.04 | beta-amylase activity |
| TraesCS3A01G181400 | 256.9 | 82.79 | 1.63 | 0.00 | 0.00 | geranylgeranyl reductase activity |
| TraesCS3B01G211400 | 248.36 | 86.7 | 1.51 | 0.00 | 0.00 | geranylgeranyl reductase activity |
| TraesCS4A01G063200 | 78.91 | 27.48 | 1.52 | 0.00 | 0.02 | UDP-3-O-[3-hydroxymyristoyl]  N-acetylglucosamine deacetylase activity |
| TraesCS1D01G341100 | 1124.17 | 549.56 | 1.03 | 0.00 | 0.03 | alpha-trehalose-phosphate synthase (UDP-forming) |
| TraesCS5A01G248900 | 35.16 | 7.05 | 2.32 | 0.00 | 0.02 | malate transmembrane transport |
| TraesCS5B01G484700 | 18.97 | 1.35 | 3.91 | 0.00 | 0.03 | Glucose-1-phosphate adenylyltransferase |
| TraesCS5D01G064100 | 1897.26 | 766.05 | 1.31 | 0.00 | 0.00 | Glucan 1,4-beta-glucosidase |
| TraesCS5D01G102600 | 59.83 | 11.62 | 2.35 | 0.00 | 0.00 | beta-1,4-mannosyltransferase activity |
| TraesCS6A01G245100 | 625.41 | 227.97 | 1.46 | 0.00 | 0.01 | carbohydrate transmembrane transporter activity |
| TraesCS6A01G148000 | 399.05 | 187.22 | 1.09 | 0.00 | 0.01 | D-arabinono-1,4-lactone oxidase activity |
| TraesCS6B01G468600 | 425.09 | 114 | 1.90 | 0.00 | 0.00 | UDP-glucosyltransferase activity |
| TraesCS6D01G239700 | 151.81 | 60.19 | 1.34 | 0.00 | 0.01 | 2-C-methyl-D-erythritol 2,4-cyclodiphosphate synthase |
| TraesCS7A01G009200 | 449.11 | 187.31 | 1.26 | 0.00 | 0.02 | 1,2-beta-fructan 1F-fructosyltransferase activity |
| TraesCS7B01G067000 | 3228 | 773.48 | 2.06 | 0.00 | 0.00 | hexose metabolic process |
| TraesCS7D01G155700 | 349.87 | 145.86 | 1.26 | 0.00 | 0.01 | UDP-glucosyltransferase activity |
| TraesCS1A01G364800 | 36.07 | 138.48 | -1.94 | 0.00 | 0.00 | polygalacturonase activity |
| TraesCS2D01G057000 | 24418.58 | 52897.9 | -1.12 | 0.00 | 0.00 | Glycoside hydrolase family |
| TraesCS4B01G344700 | 1477.99 | 3156.09 | -1.09 | 0.00 | 0.00 | Glycoside hydrolase |
| TraesCS6B01G118000 | 4.25 | 28.39 | -2.75 | 0.00 | 0.03 | Glycoside hydrolase |
| TraesCS4B01G143500 | 782.74 | 1740.41 | -1.15 | 0.00 | 0.02 | Xylanase inhibitor protein 1 |
| TraesCS4B01G344300 | 244.86 | 508.31 | -1.05 | 0.00 | 0.02 | beta-amylase activity |
| TraesCS4D01G339400 | 451.95 | 1000.24 | -1.15 | 0.00 | 0.00 | beta-amylase activity |
| TraesCS5B01G064000 | 65.02 | 149.9 | -1.20 | 0.00 | 0.03 | Glucan 1,3-beta-glucosidase |

**Table S2-III** Differently expressed genes between LN-I and HN-I related to carbon and nitrogen metabolism

| **Gene-ID** | **LN-I**  **Readcount** | **HN-I**  **Readcount** | **log2FoldChange** | ***p*-Value** | **Padj** |  |
| --- | --- | --- | --- | --- | --- | --- |
| TraesCS3B01G456700 | 23.44 | 143.94 | -2.58 | 0.00 | 0.00 | cysteine-type endopeptidase inhibitor activity |
| TraesCS1A01G265700 | 45.22 | 221.30 | -2.28 | 0.00 | 0.03 | serine-type endopeptidase inhibitor |
| TraesCS1A01G370100 | 202.54 | 535.83 | -1.41 | 0.00 | 0.04 | S-(hydroxymethyl)glutathione dehydrogenase activity |
| TraesCS2B01G372900 | 1535.52 | 3230.63 | -1.07 | 0.00 | 0.00 | adenosylmethionine decarboxylase activity |
| TraesCS6D01G054300 | 124.91 | 259.11 | -1.05 | 0.00 | 0.05 | GMP synthase (glutamine-hydrolyzing) activity |
| TraesCS1B01G276700 | 24.13 | 112.05 | -2.18 | 0.00 | 0.02 | serine-type endopeptidase inhibitor |
| TraesCS5A01G484800 | 73.44 | 238.10 | -1.69 | 0.00 | 0.00 | serine-type endopeptidase inhibitor |
| TraesCS3B01G456700 | 23.44 | 143.94 | -2.58 | 0.00 | 0.00 | Cysteine proteinase inhibitor |
| TraesCS3D01G034200 | 10.54 | 52.55 | -2.35 | 0.00 | 0.00 | serine-type endopeptidase inhibitor |
| TraesCS3D01G416700 | 21.80 | 300.23 | -3.76 | 0.00 | 0.00 | cysteine-type endopeptidase inhibitor activity |
| TraesCS5A01G417800 | 932.63 | 3305.10 | -1.83 | 0.00 | 0.00 | serine protease inhibitor |
| TraesCS1D01G224400 | 55.04 | 132.20 | -1.26 | 0.00 | 0.03 | protein transporter activity |
| TraesCS1A01G222800 | 36.47 | 86.85 | -1.26 | 0.00 | 0.03 | protein transporter activity |
| TraesCS3A01G149900 | 181.15 | 46.63 | 1.95 | 0.00 | 0.00 | basic amino acid transmembrane transport |
| TraesCS3B01G177000 | 73.54 | 16.58 | 2.16 | 0.00 | 0.01 | transmembrane transport |
| TraesCS3D01G219200 | 168.70 | 60.80 | 1.48 | 0.00 | 0.03 | alanine transmembrane transporter activity |
| TraesCS1B01G290600 | 72.05 | 23.35 | 1.63 | 0.00 | 0.01 | Gamma-glutamyl phosphate reductase |
| TraesCS2A01G161200 | 76.56 | 19.20 | 1.98 | 0.00 | 0.01 | Trehalose 6-phosphate phosphatase |
| TraesCS3D01G163400 | 58.68 | 6.85 | 3.13 | 0.00 | 0.01 | glycerol transmembrane transporter activity |
| TraesCS5B01G399800 | 363.42 | 148.73 | 1.29 | 0.00 | 0.00 | UDP-glucose 7-dehydrogenase activity |
| TraesCS5A01G394900 | 105.74 | 423.37 | -1.99 | 0.00 | 0.00 | UDP-glucose 6-dehydrogenase |
| TraesCS1D01G246300 | 37.43 | 137.77 | -1.91 | 0.00 | 0.01 | Isoamylase-like glucan debranching enzyme |
| TraesCS2B01G491000 | 12.03 | 50.18 | -2.12 | 0.00 | 0.00 | monophenol monooxygenase activity |
| TraesCS2D01G063900 | 743.39 | 1778.03 | -1.26 | 0.00 | 0.01 | glycerol transmembrane transporter |
| TraesCS2D01G561700 | 590.62 | 1294.00 | -1.13 | 0.00 | 0.00 | pyruvate kinase activity |
| TraesCS3A01G391500 | 692.33 | 1451.47 | -1.07 | 0.00 | 0.01 | fructose-bisphosphate aldolase activity |
| TraesCS3B01G211400 | 77.24 | 227.07 | -1.57 | 0.00 | 0.00 | geranylgeranyl reductase activity |
| TraesCS4A01G063200 | 23.71 | 72.15 | -1.62 | 0.00 | 0.01 | UDP-3-O-[3-hydroxymyristoyl]  N-acetylglucosamine deacetylase activity |
| TraesCS4A01G180200 | 187.84 | 641.36 | -1.77 | 0.00 | 0.00 | glycerol transmembrane transporter activity |
| TraesCS4B01G253300 | 73.15 | 172.02 | -1.24 | 0.00 | 0.01 | fucosidase activity |
| TraesCS5B01G012300 | 10.43 | 46.18 | -2.15 | 0.00 | 0.02 | phosphopyruvate hydratase |
| TraesCS5B01G127500 | 2.89 | 29.95 | -3.56 | 0.00 | 0.01 | sialyltransferase activity |
| TraesCS5B01G252100 | 1.74 | 28.78 | -3.95 | 0.00 | 0.00 | Cellulose synthase |
| TraesCS5D01G404300 | 96.31 | 363.24 | -1.91 | 0.00 | 0.00 | UDP-glucose 8-dehydrogenase activity |
| TraesCS6D01G196300 | 522.95 | 1278.44 | -1.29 | 0.00 | 0.00 | Glyceraldehyde-3-phosphate dehydrogenase |
| TraesCS7A01G009200 | 177.33 | 410.89 | -1.22 | 0.00 | 0.04 | beta-fructofuranosidase activity |
| TraesCS7A01G033100 | 338.74 | 959.04 | -1.51 | 0.00 | 0.01 | Cellulose synthase-like protein |
| TraesCS7D01G009400 | 122.38 | 440.72 | -1.86 | 0.00 | 0.02 | sucrose 1F-fructosyltransferase |
| TraesCS7D01G163100 | 548.86 | 1685.35 | -1.62 | 0.00 | 0.00 | aldose 1-epimerase activity |
| TraesCS7D01G316700 | 89.20 | 302.07 | -1.77 | 0.00 | 0.00 | Non-lysosomal glucosylceramidase |
| TraesCS5D01G216100 | 962.28 | 350.15 | 1.46 | 0.00 | 0.03 | guanosine-3',5'-bis(diphosphate) 3'-diphosphatase activity |
| TraesCS7D01G316700 | 89.20 | 302.07 | -1.77 | 0.00 | 0.00 | glucosylceramidase activity |

**Table S2-IV** Differently expressed genes between LN-O and HN-O related to carbon and nitrogen metabolism

| **Gene ID** | **LN-O**  **Readcount** | **HN-O**  **Readcount** | **log2FoldChange** | ***p-*Value** | **Padj** | **GO Annotation** |
| --- | --- | --- | --- | --- | --- | --- |
| TraesCS5B01G502700 | 626.92 | 232.22 | 1.44 | 0.00 | 0.00 | Anthranilate synthase |
| TraesCS5D01G503800 | 493.5 | 243.97 | 1.02 | 0.00 | 0.00 | Anthranilate synthase |
| TraesCS1A01G425100 | 706.48 | 312.38 | 1.18 | 0.00 | 0.00 | acid-amino acid ligase activity |
| TraesCS1B01G223300 | 52.35 | 13.34 | 1.95 | 0.00 | 0.05 | Proline dehydrogenase |
| TraesCS1D01G114800 | 139.81 | 60.53 | 1.22 | 0.00 | 0.02 | Tryptophan aminotransferase |
| TraesCS2D01G231100 | 301.9 | 131.6 | 1.20 | 0.00 | 0.00 | (thioredoxin) activity |
| TraesCS6B01G054200 | 19.89 | 2.2 | 3.13 | 0.00 | 0.03 | cysteine-type peptidase activity |
| TraesCSU01G182400 | 39.19 | 7.35 | 2.39 | 0.00 | 0.01 | cysteine-type peptidase activity |
| TraesCS3A01G420500 | 635.98 | 255.78 | 1.31 | 0.00 | 0.00 | serine-type endopeptidase activity |
| TraesCS3B01G455900 | 103.27 | 38.81 | 1.42 | 0.00 | 0.05 | serine-type endopeptidase activity |
| TraesCS5A01G427900 | 176.84 | 87.39 | 1.02 | 0.00 | 0.01 | 2Fe-3S iron-sulfur cluster binding domain |
| TraesCS5A01G382900 | 388.37 | 160.09 | 1.28 | 0.00 | 0.00 | sulfate adenylyltransferase activity |
| TraesCS3B01G177000 | 136.34 | 29.83 | 2.21 | 0.00 | 0.00 | L-histidine transmembrane transport |
| TraesCS3A01G149900 | 342.46 | 103.96 | 1.72 | 0.00 | 0.00 | L-histidine transmembrane transport |
| TraesCS3D01G157800 | 115.74 | 17.32 | 2.73 | 0.00 | 0.00 | basic amino acid transport |
| TraesCS4A01G215300 | 215.01 | 53.97 | 2.00 | 0.00 | 0.00 | Transmembrane amino acid transporter |
| TraesCS1A01G370100 | 109.65 | 268.14 | -1.30 | 0.00 | 0.00 | glutathione dehydrogenase activity |
| TraesCS1B01G322100 | 60.69 | 136.38 | -1.17 | 0.00 | 0.03 | cysteine-type endopeptidase inhibitor activity |
| TraesCS2A01G546300 | 0.31 | 22.24 | -6.01 | 0.00 | 0.01 | cysteine-type endopeptidase activity |
| TraesCS3A01G412900 | 2.72 | 51.51 | -4.29 | 0.00 | 0.00 | Cysteine peptidase |
| TraesCS3B01G447400 | 0.29 | 26.89 | -6.28 | 0.00 | 0.00 | Cysteine peptidase |
| TraesCS4D01G061400 | 22.99 | 80.11 | -1.81 | 0.00 | 0.01 | Cysteine peptidase |
| TraesCS3D01G407600 | 5.38 | 64.94 | -3.62 | 0.00 | 0.00 | Cysteine peptidase |
| TraesCS3D01G407700 | 1.49 | 27 | -4.32 | 0.00 | 0.00 | Cysteine peptidase |
| TraesCS3A01G421000 | 362.96 | 1132.27 | -1.64 | 0.00 | 0.00 | Cysteine proteinase inhibitor |
| TraesCS3B01G456500 | 427.33 | 1400.21 | -1.71 | 0.00 | 0.01 | cysteine-type endopeptidase inhibitor activity |
| TraesCS3D01G416500 | 204.38 | 647.06 | -1.66 | 0.00 | 0.02 | cysteine-type endopeptidase inhibitor activity |
| TraesCS3D01G416600 | 7.22 | 36.1 | -2.34 | 0.00 | 0.03 | cysteine-type endopeptidase inhibitor activity |
| TraesCS3D01G416700 | 52.69 | 551.46 | -3.39 | 0.00 | 0.00 | cysteine-type endopeptidase inhibitor activity |
| TraesCS2A01G355400 | 1984.82 | 4790.49 | -1.27 | 0.00 | 0.01 | S-adenosylmethionine decarboxylase proenzyme |
| TraesCS2D01G352900 | 2694.6 | 5710.73 | -1.08 | 0.00 | 0.01 | S-adenosylmethionine decarboxylase proenzyme |
| TraesCS7D01G063900 | 266.47 | 809.47 | -1.60 | 0.00 | 0.00 | S-adenosylmethionine decarboxylase proenzyme |
| TraesCS1A01G265800 | 473.01 | 1413.03 | -1.58 | 0.00 | 0.00 | serine-type endopeptidase inhibitor |
| TraesCS1A01G265900 | 613.76 | 1408.9 | -1.20 | 0.00 | 0.00 | serine-type endopeptidase inhibitor activity |
| TraesCS1B01G276700 | 53.61 | 183.52 | -1.78 | 0.00 | 0.00 | serine-type endopeptidase inhibitor activity |
| TraesCS1B01G276900 | 334.06 | 934.3 | -1.48 | 0.00 | 0.00 | serine-type endopeptidase inhibitor |
| TraesCS1D01G189400 | 49.43 | 279.93 | -2.51 | 0.00 | 0.00 | serine-type endopeptidase activity |
| TraesCS1D01G265900 | 740.7 | 1777.05 | -1.26 | 0.00 | 0.00 | serine-type endopeptidase inhibitor activity |
| TraesCS1D01G266100 | 115.35 | 322.63 | -1.48 | 0.00 | 0.00 | serine-type endopeptidase inhibitor |
| TraesCS5A01G526000 | 51.67 | 533.96 | -3.38 | 0.00 | 0.00 | serine-type endopeptidase activity |
| TraesCS5B01G404600 | 152.26 | 431.4 | -1.50 | 0.00 | 0.00 | serine-type endopeptidase activity |
| TraesCS5B01G478200 | 161.52 | 523.18 | -1.70 | 0.00 | 0.00 | serine-type endopeptidase inhibitor |
| TraesCS4A01G176100 | 37.34 | 119.36 | -1.67 | 0.00 | 0.00 | serine-type endopeptidase activity |
| TraesCS7D01G466800 | 90.09 | 231.96 | -1.37 | 0.00 | 0.03 | Ureide permease |
| TraesCS7B01G382000 | 34.73 | 122.35 | -1.82 | 0.00 | 0.00 | Ureide permease |
| TraesCS7A01G376800 | 46.13 | 132.01 | -1.52 | 0.00 | 0.03 | Thiamine thiazole synthase |
| TraesCS5D01G407800 | 69.18 | 153.78 | -1.16 | 0.00 | 0.04 | Aspartate-semialdehyde dehydrogenase |
| TraesCS5A01G428200 | 144.45 | 429.32 | -1.57 | 0.00 | 0.00 | 2Fe-2S iron-sulfur cluster binding domain |
| TraesCS7A01G169100 | 47.25 | 351.97 | -2.90 | 0.00 | 0.00 | EamA-like transporter family |
| TraesCS2B01G423700 | 127.26 | 418.01 | -1.71 | 0.00 | 0.01 | Transmembrane amino acid transporter |
| TraesCS6B01G007400 | 1.92 | 25.93 | -3.75 | 0.00 | 0.00 | Transmembrane amino acid transporter |
| TraesCS1D01G067800 | 895.58 | 442.35 | 1.02 | 0.00 | 0.00 | acyl-CoA dehydrogenase activity |
| TraesCS2B01G341200 | 712.61 | 351.27 | 1.02 | 0.00 | 0.00 | Aldose 1-epimerase |
| TraesCS2D01G166600 | 292.02 | 138.11 | 1.09 | 0.00 | 0.04 | glycogen debranching enzyme activity |
| TraesCS3A01G159900 | 323.24 | 148.31 | 1.13 | 0.00 | 0.00 | beta-amylase activity |
| TraesCS3D01G167100 | 94.57 | 36.36 | 1.39 | 0.00 | 0.04 | beta-amylase activity |
| TraesCS4A01G307900 | 252.66 | 105.23 | 1.27 | 0.00 | 0.00 | beta-amylase activity |
| TraesCS5B01G556500 | 73.87 | 16.24 | 2.17 | 0.00 | 0.00 | xyloglucan:xyloglucosyl transferase activity |
| TraesCS5D01G403700 | 1874.77 | 580.41 | 1.69 | 0.00 | 0.00 | UDP-glucoronosyl and UDP-glucosyl transferase |
| TraesCS7A01G189000 | 413.87 | 157.6 | 1.39 | 0.00 | 0.00 | UDP-glucosyltransferase activity |
| TraesCS7A01G215900 | 658.91 | 302.79 | 1.12 | 0.00 | 0.02 | UDP-glucoronosyl and UDP-glucosyl transferase |
| TraesCS6D01G227500 | 1216.01 | 453.79 | 1.42 | 0.00 | 0.04 | glycerol transmembrane transporter activity |
| TraesCS6A01G319300 | 1.32 | 33.89 | -4.71 | 0.00 | 0.00 | amylase activity |
| TraesCS1B01G068600 | 4.26 | 39.13 | -3.17 | 0.00 | 0.00 | UDP-glucose transmembrane transporter activity |
| TraesCS1B01G374500 | 81.63 | 175.75 | -1.11 | 0.00 | 0.01 | 3-chloroallyl aldehyde dehydrogenase activity |
| TraesCS2B01G244600 | 27.52 | 168.76 | -2.62 | 0.00 | 0.00 | Isocitrate lyase |
| TraesCS2D01G224200 | 23.54 | 127.57 | -2.46 | 0.00 | 0.00 | Isocitrate lyase |
| TraesCS3B01G211400 | 20.32 | 94.58 | -2.21 | 0.00 | 0.00 | geranylgeranyl reductase activity |
| TraesCS4A01G393500 | 83.41 | 180.55 | -1.12 | 0.00 | 0.03 | Acyl-CoA-binding protein |
| TraesCS4B01G143500 | 837.39 | 1898.44 | -1.18 | 0.00 | 0.01 | Glycoside hydrolase |
| TraesCS4D01G187500 | 2.18 | 21.53 | -3.41 | 0.00 | 0.02 | mannosyl-oligosaccharide 1,2-alpha-mannosidase activity |
| TraesCS4B01G220200 | 95.85 | 268.63 | -1.49 | 0.00 | 0.02 | phosphoenolpyruvate carboxykinase activity |
| TraesCS4D01G220500 | 21.99 | 62.79 | -1.52 | 0.00 | 0.04 | Phosphoenolpyruvate carboxykinase |
| TraesCS5A01G305100 | 95.18 | 205.97 | -1.12 | 0.00 | 0.04 | UDP-glucoronosyl and UDP-glucosyl transferase |
| TraesCS5A01G394900 | 245.73 | 491.33 | -1.00 | 0.00 | 0.01 | UDP-glucose 6-dehydrogenase activity |
| TraesCS5A01G138400 | 475.51 | 966.34 | -1.02 | 0.00 | 0.00 | alpha-L-arabinofuranosidase activity |
| TraesCS5B01G252100 | 4.47 | 33.98 | -2.98 | 0.00 | 0.00 | cellulose synthase (UDP-forming) activity |
| TraesCS5D01G312100 | 53.52 | 128.65 | -1.27 | 0.00 | 0.05 | UDP-glucoronosyl and UDP-glucosyl transferase |
| TraesCS5D01G404300 | 120.65 | 365.57 | -1.60 | 0.00 | 0.00 | UDP-glucose 6-dehydrogenase |
| TraesCS7D01G009400 | 17.47 | 118.63 | -2.79 | 0.00 | 0.00 | sucrose 1F-fructosyltransferase activity |
| TraesCS6A01G093200 | 132.01 | 341.23 | -1.37 | 0.00 | 0.00 | Glycoside hydrolase |
| TraesCS7D01G351300 | 63.56 | 371.48 | -2.55 | 0.00 | 0.00 | Glycosyl hydrolases |
| TraesCS6B01G342600 | 246.2 | 678.99 | -1.46 | 0.00 | 0.00 | Glycoside hydrolase |
| TraesCS6D01G291800 | 288.44 | 698.17 | -1.28 | 0.00 | 0.00 | Glycoside hydrolase |
| TraesCS7A01G371600 | 341.46 | 1064.17 | -1.64 | 0.00 | 0.00 | Glycosyl hydrolases |
| TraesCS7B01G256000 | 46.71 | 132.42 | -1.51 | 0.00 | 0.03 | Glycosyl hydrolases |
| TraesCS6A01G334100 | 1.12 | 38.61 | -5.28 | 0.00 | 0.00 | Alpha-amylase |
| TraesCS6A01G334200 | 57.23 | 357.96 | -2.65 | 0.00 | 0.00 | Alpha-amylase |
| TraesCS6D01G298500 | 3.12 | 22.54 | -2.83 | 0.00 | 0.02 | Alpha-amylase |
| TraesCS6D01G313500 | 3.84 | 92.67 | -4.66 | 0.00 | 0.00 | Alpha-amylase |
| TraesCS7A01G033100 | 202.62 | 414.44 | -1.03 | 0.00 | 0.00 | cellulose synthase |
| TraesCS7A01G298600 | 312.14 | 1467.43 | -2.23 | 0.00 | 0.00 | cellulose synthase |
| TraesCS7B01G188400 | 847.48 | 3051.93 | -1.85 | 0.00 | 0.00 | cellulose synthase |
| TraesCS6D01G392100 | 193.11 | 419.7 | -1.12 | 0.00 | 0.00 | Xyloglucan endotransglucosylase |
| TraesCS7A01G427000 | 2.86 | 20.39 | -2.83 | 0.00 | 0.04 | Xyloglucan endotransglucosylase |
| TraesCS7D01G419900 | 3.12 | 74.96 | -4.56 | 0.00 | 0.00 | Xyloglucan endotransglucosylase |
| TraesCS7D01G163100 | 30.56 | 184.04 | -2.60 | 0.00 | 0.00 | Aldose 2-epimerase |
| TraesCS6D01G130800 | 40.52 | 140.08 | -1.79 | 0.00 | 0.02 | 4-hydroxyphenylpyruvate dioxygenase |
| TraesCS4A01G180200 | 145.08 | 385.32 | -1.41 | 0.00 | 0.00 | glycerol transmembrane transporter activity |
