# Supplementary material for: Transcriptomic and Metabolomics Analysis of Different Endosperm Region under Nitrogen Treatments
Source: Int J Mol Sci. 2019 Aug 28;20(17):4212. doi: 10.3390/ijms20174212 (PMC6747615; doi:10.3390/ijms20174212)
Supplement: Supplementary file 1 [file ijms-20-04212-s001.zip › ijms-575327-for final-suppl/Supplementary-Table S3.doc]

**Table S3.** PCA loading scores of the identified metabolites in this study

| **Metabolites** | **V1** | **V2** |
| --- | --- | --- |
| L-Saccharopine | 0.79 | 0.39 |
| L-Glutamine | 0.93 | -0.11 |
| 1-Aminocyclopropanecarboxylic acid | 0.93 | -0.09 |
| Trigonelline | 0.91 | -0.03 |
| Dopamine | 0.90 | 0.10 |
| Allantoin | 0.92 | -0.28 |
| Maltotriose | 0.90 | -0.36 |
| Hypoxanthine | 0.88 | 0.22 |
| L-Tyrosine | 0.88 | 0.14 |
| 1,2,3-Benzenetriol | 0.88 | -0.28 |
| Indoleacetic acid | -0.85 | -0.23 |
| O-Acetyl-L-serine | 0.75 | -0.07 |
| Tyramine | 0.78 | -0.36 |
| Inosine | 0.86 | 0.22 |
| L-Aspartate | -0.73 | 0.55 |
| L-Serine | -0.57 | 0.55 |
| Maltopentaose | -0.13 | -0.07 |
| L-Phenylalanine | 0.78 | -0.37 |
| myo-Inositol | 0.77 | -0.51 |
| DL-2-Aminoadipic acid | 0.15 | 0.84 |
| Choline | 0.64 | -0.08 |
| 4-Guanidinobutyric acid | 0.86 | -0.28 |
| Pyridoxine | 0.71 | -0.30 |
| 1-Palmitoyl-2-hydroxy-sn-glycero-3-phosphoethanolamine | 0.53 | 0.76 |
| Sucrose | 0.80 | -0.40 |
| Thiamine | -0.29 | 0.05 |
| DL-Arginine | 0.72 | 0.20 |
| Raffinose | -0.31 | -0.77 |
| 1-Stearoyl-sn-glycerol 3-phosphocholine | 0.38 | 0.62 |
| 1-Palmitoylglycerol | -0.24 | 0.65 |
| Adenosine | -0.46 | -0.04 |
| Glycerophosphocholine | 0.41 | 0.82 |
| Thymidine | -0.08 | -0.28 |
| Betaine | -0.67 | -0.48 |
| 2-Ethoxyethanol | 0.16 | -0.26 |
| Phosphorylcholine | 0.76 | -0.22 |
| Thioetheramide-PC | -0.51 | 0.70 |
| D-Glucose 6-phosphate | -0.14 | -0.38 |
| Nicotinate | -0.39 | -0.50 |
| Cellobiose | -0.48 | -0.67 |
| Isomaltose | -0.45 | -0.49 |
| 1-Oleoyl-sn-glycero-3-phosphocholine | 0.23 | 0.56 |
| L-Methionine | 0.35 | 0.74 |
| Uracil | 0.32 | 0.04 |
| L-Pipecolic acid | 0.33 | -0.34 |
| Galactinol | -0.26 | -0.40 |
| 1-Stearoyl-2-oleoyl-sn-glycerol 3-phosphocholine (SOPC) | -0.50 | 0.66 |
| Mannobiose | -0.36 | -0.56 |
| L-Pyroglutamic acid | 0.42 | 0.72 |
