# Supplementary material for: Transcriptomic and Metabolomics Analysis of Different Endosperm Region under Nitrogen Treatments
Source: Int J Mol Sci. 2019 Aug 28;20(17):4212. doi: 10.3390/ijms20174212 (PMC6747615; doi:10.3390/ijms20174212)
Supplement: Supplementary file 1 [file ijms-20-04212-s001.zip › ijms-575327-for final-suppl/Supplementary-Table S4.doc]

**Table S4.** Oligonucleotide primer used in this study

| **Gene ID** | **Prime 5′-3′** |
| --- | --- |
| TraesCS2B01G294500 | F:ATCTTGCTGTGGTTCCTCCTAA |
|  | R:CGCATTTCCTTCATCTTTCTTT |
| TraesCS2D01G536300 | F:GTGCGTGGTGCTTGTAGTGG |
|  | R:TGTCTTGAGGAAGTTGGGGC |
| TraesCS5D01G431100 | F:CAAGGGTGACCGACAGGACA |
|  | R:ACGCCAGAGGAGGGAAAGTG |
| TraesCS2D01G383600 | F:AAGGAGCGGAAGGAGAGGAA |
|  | R:TGGCGAGTTTAGCAAGAGCA |
| TraesCS4A01G52100 | F:CTTCTTCTCCTTGCTGCTCTTG |
|  | R:AGTTCATGCCCTCAGTCATTCTC |
| TraesCS5B01G400600 | F:ATGCCCCGCCATAACTTCTC |
|  | R:CGTTTGTCCACCTCCACCAC |
| TraesCS7D01G448200 | F:CTTCCTAGTCCTTGTGGCTCTCTC |
|  | R:CAGTCTCGCATCACCTTTTTTTTA |
| Actin | F: CCTCTCTTAGCACTTTCCAGCA |
|  | R:GTAAGTCCCCTTCACCGACTC |
